# Computational and experimental assessment of key interdomain residues controlling the fold‐switch of RfaH

**DOI:** 10.1002/pro.70202

**Published:** 2025-06-16

**Authors:** Cyndi Tabilo‐Agurto, Javiera Reyes, Irina Artsimovitch, César A. Ramírez‐Sarmiento

**Affiliations:** ^1^ Institute for Biological and Medical Engineering, Schools of Engineering, Medicine and Biological Sciences Pontificia Universidad Católica de Chile Santiago Chile; ^2^ ANID, Millennium Science Initiative Program Millennium Institute for Integrative Biology (iBio) Santiago Chile; ^3^ Department of Microbiology and Center for RNA Biology The Ohio State University Columbus Ohio USA

**Keywords:** in vivo assay, metamorphic proteins, protein structure prediction, structure‐based models

## Abstract

*Escherichia coli* RfaH, a member of the universally conserved NusG family of transcription factors, regulates its function by undergoing a structural rearrangement of its C‐terminal domain (CTD) upon recruitment to RNA polymerase (RNAP) paused at the DNA signal known as *operon polarity suppressor* (*ops*) element. While it is known that the fold‐switch of RfaH CTD from an α‐helical hairpin (autoinhibited state) into a β‐barrel (active state) is controlled by interactions between the N‐terminal domain (NTD) and CTD, which are broken apart upon NTD binding to RNAP, a comprehensive analysis of residues that stabilize the autoinhibited state is lacking. Here, we utilize a combination of molecular dynamics (MD), protein structure prediction, and in vivo functional assays as a workflow to determine key interdomain (ID) residues controlling the fold‐switch of RfaH. First, MD simulations employing structure‐based models identified eight CTD residues with high ID contact probability, therefore expected to play a crucial role in stabilizing the autoinhibited state of RfaH. In silico alanine scanning mutagenesis followed by structure prediction using AlphaFold2 showed that four of these mutants (F126A, E136A, R138A, and L142A) led to several models with mixed α/β secondary structure for the CTD in comparison to the known fold‐switching mutant E48A. Lastly, experimental alanine scanning mutagenesis and RfaH‐dependent in vivo luminescence assays confirmed that I129 and L142 contribute to the stabilization of the autoinhibited state. These results deepen our understanding of the fold‐switch of RfaH, with tools that are applicable to other metamorphic proteins.

## 1 INTRODUCTION

Nearly 100 proteins have been experimentally determined to undergo fold‐switching (Porter & Looger, 2018), a phenomenon of drastic change in secondary, tertiary, and (sometimes) quaternary structure between two native states, often with different biological functions, encoded by a single amino acid sequence (Porter et al., 2024). These often called metamorphic proteins participate in crucial cellular processes, such as regulation of the cyanobacterial circadian clock (Chang et al., 2015), inflammatory responses (Tuinstra et al., 2008), and ion transport (Littler et al., 2004).

**FIGURE 1:**
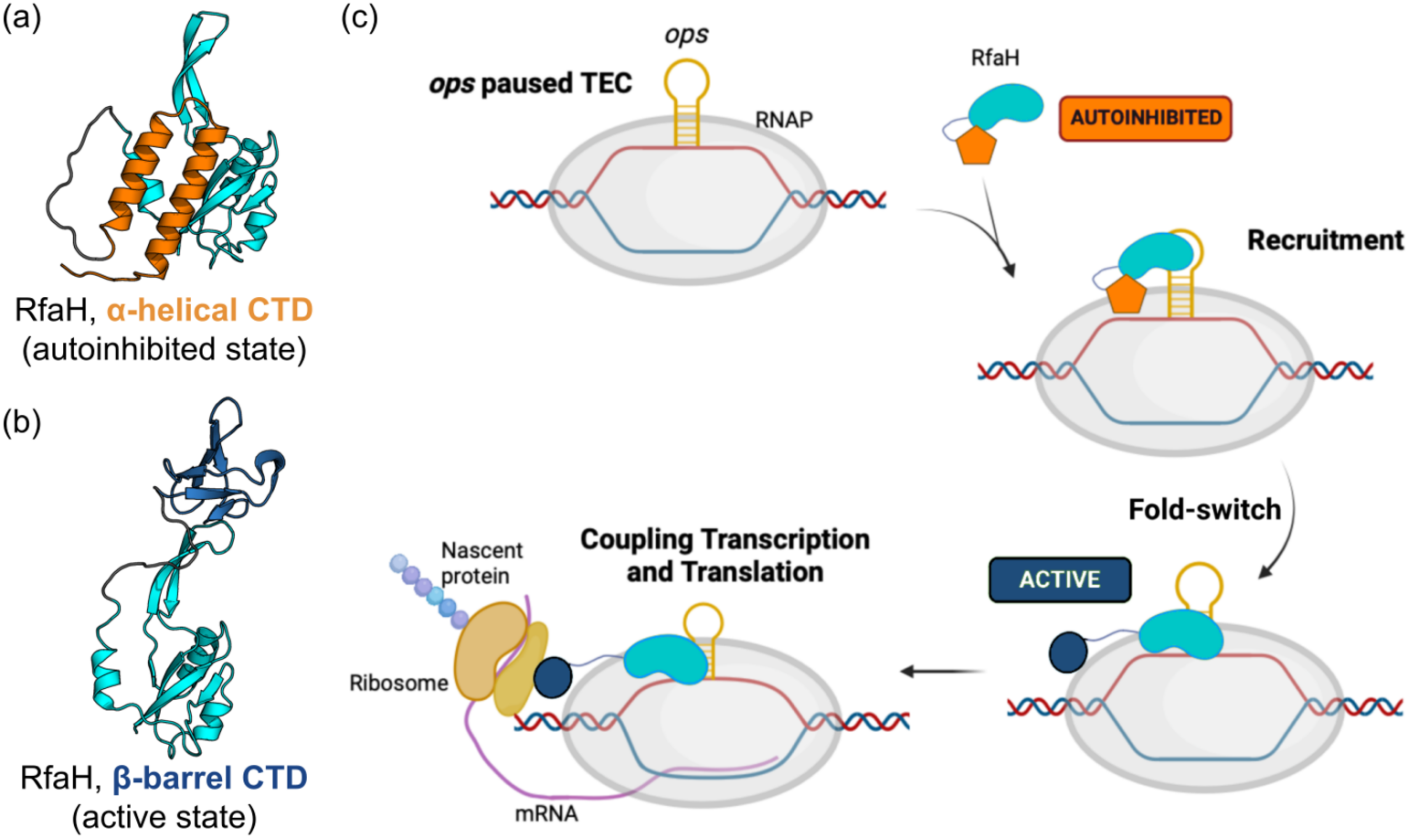
Fold‐switch and function of RfaH. (a) Structure of the autoinhibited state of RfaH, modeled in this work based on Protein Data Bank (PDB) identification codes 2OUG and 5OND. The N‐terminal domain (NTD) is shown in cyan, and the α‐helical C‐terminal domain (CTD) is shown in orange. (b) Structure of the active state of RfaH (chain D of PDB 6C6S), with the β‐barrel CTD in blue. (c) Scheme of the mechanism of action of RfaH. Autoinhibited RfaH is recruited by the exposed *operon polarity suppressor* (*ops*) DNA signal in the non‐template strand of paused transcription elongation complexes (TEC). Upon recruitment, RfaH fold‐switches into the active state to couple transcription and translation of virulence genes by binding to the RNA polymerase (RNAP) via its NTD and to the ribosome via its CTD.

*Escherichia coli* RfaH, a paralog of housekeeping NusG, the only universally conserved transcription factor, is a quintessential example of a metamorphic protein (Artsimovitch & Ramírez‐Sarmiento, 2022). RfaH is required for the expression of genes associated with virulence and is specifically recruited to RNA polymerase (RNAP) paused at a 12‐nt long DNA sequence known as *ops* element (Artsimovitch & Landick, 2002). Upon recruitment, RfaH switches from an autoinhibited state, in which its C‐terminal Kyprides, Ouzounis, Woese (KOW) domain (C‐terminal domain [CTD]) is folded as an α‐helical hairpin tightly bound to the N‐terminal domain (NTD), into an active state in which the CTD dissociates from the NTD and refolds into a β‐barrel (Burmann et al., 2012). This dissociation and fold‐switching enables the NTD to bind to RNAP to promote processive RNA synthesis, whereas the refolded CTD binds to the ribosome to couple transcription and translation (Molodtsov et al., 2024; Zuber et al., 2024) (Figure 1).

Computationally, comparative analysis of over a thousand sequences of RfaH and of NusG, in which the NTD and CTD do not interact (Wang et al., 2020), uncovered two residues that, when changed to the corresponding amino acid identities in NusG (I93E, F130V), locked RfaH in the active state (Shi et al., 2017). One of these residues, F130, was already suggested by molecular dynamics (MD) simulations using simplified coarse‐grained structure‐based models (SBMs), that is, models that incorporate distance‐based residue pair contacts implicitly in the potential energy function of the simulation system, to be important for the fold‐switch of RfaH (Ramírez‐Sarmiento et al., 2015).

Additionally, protein structure predictions of full‐length RfaH using AlphaFold2 (Jumper et al., 2021), informed by the evolutionary conservation of local energetic frustration across many RfaH sequences and structures, identified the interdomain (ID) interacting CTD residue L142 as another important residue controlling the fold‐switch of RfaH (Freiberger et al., 2023), although without experimental validation.

As shown through a combination of MD simulations and hydrogen‐deuterium exchange mass spectrometry experiments, both for the full‐length RfaH protein folded into the autoinhibited state and for the isolated CTD folded in the β‐barrel conformation, the most stable region of RfaH CTD in the autoinhibited state corresponds to the tip of the α‐helical hairpin (residues 125–145) due to persistent interactions with the NTD (Galaz‐Davison et al., 2020). The stability of this region and the melting of the ends of the α‐helical hairpin of the CTD were further confirmed by the cryo‐electron microscopy (cryoEM) analysis of the initial recruitment of C51‐C139 disulfide bridged RfaH, which is locked in the autoinhibited state, to the *ops* paused transcription elongation complexes (TEC), and its subsequent activation through domain dissociation (Zuber et al., 2024). These results indicate that other RfaH residues, beyond I93 and F130, also control its fold‐switch.

In this work, we employed MD simulations utilizing all‐atom SBMs (i.e., atomistic models informing atom‐pair distance‐based contacts in the potential energy function) (Whitford et al., 2009), protein structure predictions using AlphaFold2 through ColabFold (Mirdita et al., 2022), and functional in vivo luminescence assays (Burmann et al., 2012) to determine other CTD residues that control the fold‐switch of RfaH. Our MD simulations determined that residues I129, E136, R138, S139, L142, L143, I146, and N147 are important for the ID interaction stability. Feeding alanine scanning mutants of these residues into AlphaFold2 for protein structure prediction further confirmed that residues F126, E136, R138, and L142 might play a role in RfaH fold‐switching. By carefully selecting a subset of these residues for experimental alanine scanning and in vivo functional assays, ascertaining RfaH cellular activity, we validated that I129A and L142A dramatically activate RfaH independently of the *ops* signal. Our results further demonstrate that several ID interface residues located at the tip of the α‐helical hairpin cooperate in stabilizing the autoinhibited state of RfaH.

## 2 RESULTS

### 2.1 All‐atom SBM simulations identify residues with high interdomain contact probability

We started by developing all‐atom SBMs for the full‐length RfaH protein, generated based on the crystal structures of RfaH in the autoinhibited state (see Section 4). A key difference with previous works from our group (González‐Higueras et al., 2024) is that we utilized a single‐basin model, that is, capturing the native contacts of the autoinhibited state of RfaH only, rather than a dual‐basin model that can reversibly interconvert between the autoinhibited and active states of RfaH by incorporating both native contact maps into the model (Retamal‐Farfán et al., 2023). This approach was chosen because we wanted to capture the key residues at the NTD–CTD interface instead of the full refolding process of RfaH.

All‐atom SBM‐based MD simulations of RfaH were performed at different temperatures below and above the folding temperature of the system, to observe several ID binding and unbinding events alongside the different trajectories, as well as the unfolding of the NTD and CTD (Figure S1). We then combined the configurations explored in these trajectories, ascertained by determining the total number of residue‐residue native contacts of the system (Q_all_), alongside the potential energy of each configuration, to determine the folding temperature of our simulation system using the weighted histogram analysis method (WHAM) (Kumar et al., 1992).

**FIGURE 2:**
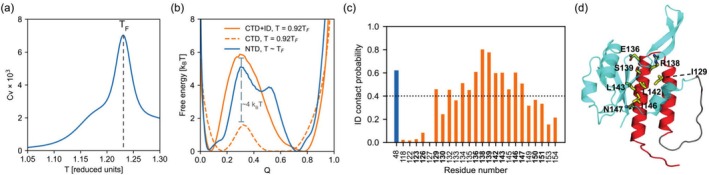
The folding landscape of RfaH and interdomain (ID) contact probabilities of C‐terminal domain (CTD) residues. (a) Heat capacity of full‐length RfaH in the autoinhibited state as a function of temperature. The heat capacity peak corresponding to the folding temperature (T_*F*_) of RfaH is indicated by a dashed line. (b) Free energy landscapes for the N‐terminal domain (NTD) and CTD of RfaH using different reaction coordinates: The sum of Q_CTD_ and Q_ID_ (solid orange line), Q_CTD_ alone (dashed orange line), and Q_NTD_ (solid blue line). The temperatures at which these free energy landscapes were obtained are indicated in the figure, and correspond to 0.92T_*F*_ for Q_CTD_ and the sum of Q_CTD_ and Q_ID_, and T_*F*_ for Q_NTD_. The dashed gray line indicates the difference in the free energy barrier for the unfolding of the CTD upon addition of Q_ID_ (~4 k_B_T). (c) ID contact probabilities for CTD residues that form ID contacts with the NTD. The residue numbers in bold correspond to those whose side chains are located less than 4 Å away from the NTD. The contact probability of the NTD residue E48, which is known for its role in RfaH fold‐switching, is indicated in blue. (d) Cartoon representation of RfaH in the autoinhibited state, with CTD residues having ID contact probabilities >0.4 shown as yellow sticks. RfaH NTD is shown in cyan over the α‐helical CTD, which is shown in red. The linker connecting the NTD and CTD domains (residues 101–114) is shown in gray.

The heat capacity of our simulated system has one peak, corresponding to the folding temperature (T_*F*_), centered at ~1.23 reduced temperature units, with an onset of unfolding starting at ~1.10 reduced temperature units (Figure 2a). By splitting the total number of residue pair native contacts into those corresponding to the NTD (Q_NTD_), CTD (Q_CTD_), and ID (Q_ID_) interactions, we can better describe the unfolding events of RfaH at different temperatures.

It is at this heat capacity onset, at T = 0.92 T_*F*_, that the domain dissociation and RfaH CTD unfolding occur. As can be seen in the free energy landscape projected in Figure 2b, not only is dissociation concurrent with CTD unfolding (Figure S1), but also the ID interactions contribute ~4 k_B_T of stability to the folding of the CTD, as observed by comparing the height of the free energy barrier when using Q_CTD_ and the sum of Q_CTD_ and Q_ID_ as reaction coordinates to determine the free energy landscape of RfaH CTD (Figure 2b). In contrast, RfaH NTD unfolding occurs near T_*F*_. These results, indicating that unfolding and dissociation of the CTD occur before NTD unfolding, are consistent with previous simulations of full‐length RfaH in the autoinhibited state in explicit solvent using steered MD (Gc et al., 2015) and temperature unfolding and steered MD simulations using a physics‐based implicit solvent model for Monte Carlo simulations of protein folding (Seifi et al., 2021).

We then calculated the residue pair ID contact probabilities of all CTD residues at the transition state of NTD–CTD dissociation and CTD unfolding observed in Figure 2b (*Q* ~0.35) from the simulation trajectories obtained at T = 0.92 T_*F*_ (Figure 2c). The rationale behind the decision to determine these contact probabilities only for CTD residues is based on our experimental design, in which RfaH NTD binds to RNAP to activate transcription of a luminescence reporter (Belogurov et al., 2010). In such a scenario, alanine scanning substitutions of NTD residues participating in ID interactions may not only induce CTD dissociation, but could also be detrimental for RfaH activity in vivo, as shown previously (Belogurov et al., 2010).

As expected from prior experimental and computational evidence indicating that the tip of the α‐helical hairpin (residues 125–145) is the most stable region of the CTD in the autoinhibited state (Galaz‐Davison et al., 2020; Ramírez‐Sarmiento et al., 2015; Seifi et al., 2021), we observe that residues 129–147 exhibit the highest contact probabilities, above 0.4 (Figure 2c). In comparison, the contact probability of the NTD ID residue E48 is ~0.6. Thus, we selected residues with an ID contact probability above 0.4 to guide our protein structure prediction and experimental validation. To further reduce the number of residues for computational and experimental alanine scanning mutagenesis, we only chose those CTD residues whose side chains are less than 4 Å away from the NTD. These residues correspond to I129, E136, R138, S139, L142, L143, I146, and N147 (Figure 2d). Of these residues, R138 is known to form a salt bridge with E48 (Burmann et al., 2012), whereas L142 was previously determined to be relevant for the fold‐switch of RfaH based on evolutionary analysis of the conservation of local energetic frustration (Freiberger et al., 2023).

### 2.2 AlphaFold2 predictions confirm the relevance of CTD residues for RfaH fold‐switch

We further evaluated the role of these residues for the fold‐switch of RfaH by using ColabFold (Mirdita et al., 2022), a cloud‐computing version of AlphaFold2 (Jumper et al., 2021), as a predictor of the potential effect of these mutations on the conformational diversity of full‐length RfaH. While researchers have shown that caution must be taken in the use of AlphaFold2 as an oracle of structure prediction for metamorphic proteins (Chakravarty & Porter, 2022), recent works have demonstrated that AlphaFold2 can predict local structural changes caused by single mutations in several proteins (McBride et al., 2023) and that evolution‐informed and physics‐informed mutations (i.e., based on the principles of local energetic frustration) in the input sequence used in AlphaFold2 can indeed drive the prediction of a few metamorphic proteins toward their different folds (Freiberger et al., 2023; Guan et al., 2024).

Thus, we used ColabFold to perform protein structure predictions for the full‐length sequence of wild‐type (WT) RfaH and eight alanine scanning variants chosen based on our all‐atom SBM‐based MD simulations (I129A, E136A, R138A, S139A, L142A, L143A, I146A, and N147A), as well as the E48A variant as a positive control and two residues with low contact probability (F126, V154) that were randomly chosen as negative controls. To increase the sampling of predicted structures using AlphaFold2, and to test the relevance of iterative recycling and the use of multiple sequence alignments (MSAs) derived for each RfaH variant by MMseqs2 in ColabFold (Steinegger & Söding, 2017) or a single MSA as the one retrieved for WT RfaH, we utilized different combinations of parameters that are significantly different from the default parameters in ColabFold.

First, we increased the number of random seeds to 10, which, considering the five model parameters available from AlphaFold2, leads to generating a total of 50 predicted structures per input sequence. In all cases, predictions were made without using structural templates. Second, we tested two different regimes of iterative refinement of the predicted structures through the AlphaFold architecture: 3 recycles and 12 recycles. Third, we ran predictions with and without dropout layers in the neural network, as it has been shown that this dropout strategy increases the structural diversity of the sampled models (Wallner, 2023). Finally, we tested if the use of MSAs retrieved from MMseqs2 for each RfaH variant independently gave different results in the predicted structures when compared to using the same MSA, as the one generated for WT RfaH, to perform the structure predictions of all variants.

Altogether, this analysis generated 4800 predicted structures, comprising 600 predicted structures per set of parameters for all 12 RfaH variants. Given the large number of predicted structures to analyze, we also performed different methods of clustering to distinguish between predicted RfaH structures that either comprised an all‐α‐helical hairpin in the CTD or a mixture of α‐helical and β‐strand secondary structures. Visual inspection of all structures showed two important features for our choice of clustering: (i) the presence of β‐strand secondary structure content was only found between residues 114–131; and (ii) the orientation of the CTD with respect to the NTD showed fluctuations between different predicted structures.

Based on this prior information, we first implemented a *k*‐means clustering approach based on the root mean square deviation (RMSD) of the Cα of residues 126–131, which easily distinguished between α‐helical and β‐strand secondary structures for this region. Since all best predicted structures (Rank 1) for all RfaH variants in all the different parameter conditions tested in ColabFold unambiguously generated an all‐α CTD, a higher RMSD for residues 126–131 sufficiently described variants with β‐strand secondary structure.

When using 3 recycles and 10 random seeds, irrespective of the use of dropouts, we were able to group the predicted structures of all RfaH variants into two clusters, based on the elbow method for determining the optimal number of clusters (Figures S2–S5). Upon analyzing the predicted structures in each cluster when dropouts were disabled, we observed a similar or higher number of predicted structures with mixed α/β secondary structure content in the CTD for three out of the eight RfaH CTD alanine scanning variants with high ID contact probability (R138A, L142A, and I146) when compared to the E48A control (Figure S6), which is known to destabilize the autoinhibited state of RfaH and lead to reversible fold‐switching (Burmann et al., 2012). In these predicted structures, residues 114–131 formed a two‐stranded antiparallel β‐sheet whereas residues 135–158 formed an α‐helix, as shown by the representative structures from each cluster in Figures S3 and S5. Upon enabling dropout layers in the neural network, we observed both an increase in the number of predicted models with mixed α/β content in the CTD for variants E48A, F126A, E136A, R138A, S139A, L143A, I146A, and N147A, with R138A and L142A showing the highest number of structures with mixed α/β secondary structure content in the CTD, as well as a propensity for some of the representative structures of these clusters to exhibit rotations of the CTD relative to the NTD (Figure S5).

**FIGURE 3:**
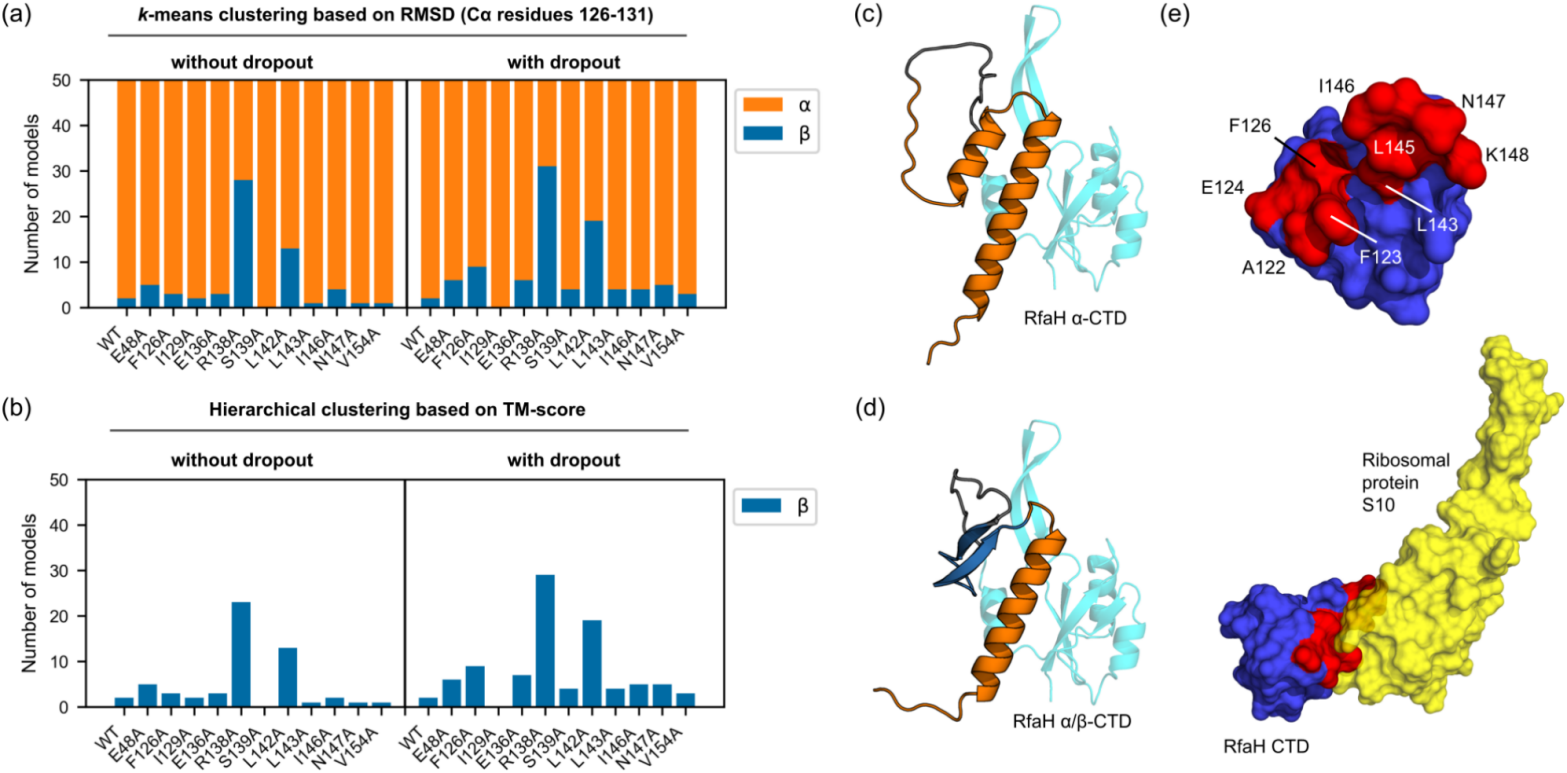
ColabFold predictions ascertain C‐terminal domain (CTD) residues behind RfaH fold‐switching. (a) Counting of the number of RfaH models generated by ColabFold for each variant exhibiting either α‐helical content (orange), or a β‐strand content (blue), as ascertained using *k*‐means clustering based on the root mean square deviation (RMSD) of the Cα of CTD residues 126–131 against the best predicted structure for each variant. ColabFold was run without using structural templates, using 10 random seeds, five model parameters and 12 recycles, thus generating 50 models per input sequence, either without or with dropouts. (b) Counting the number of RfaH models with β‐strand content (blue) for CTD residues 114–131, as ascertained by hierarchical clustering based on a dissimilarity distance matrix based on the Template Modeling (TM)‐score of each predicted structure against the best predicted structure for each variant. The dissimilarity score corresponded to 1—TM‐score. (c) Representative predicted structure of RfaH L142A with a fully α‐helical CTD (orange). The NTD (residues 1–100) is shown in cyan. (d) Representative predicted structure of RfaH L142A with a mixed α/β‐folded CTD, with the α‐helical region in orange (residues 132–158) and the β‐stranded region (residues 114–131) in blue. The NTD (residues 1–100) is shown in cyan. (e) Surface representation of the RfaH CTD (blue), highlighting the residues that interact (red) with the ribosomal protein S10 (yellow) in coupled transcription‐translation complexes when RfaH is in the active state (PDB 8UQP).

Given that increasing the number of recycles improves protein structure prediction for difficult targets (Mirdita et al., 2022), we ran ColabFold again but increased the number of recycles to 12 (Figures S7–S10). Upon doing so, only RfaH variants R138A and L142A showed a higher number of predicted structures with mixed α/β CTD secondary structure content than E48A, and only residues F126A and E136A showed a slight increase in the number of predicted structures with mixed α/β‐folded CTD with dropouts activated (Figure 3a). Thus, our results suggest that F126A, E136A, R138A, and L142A lead to mixed predicted structures irrespective of the number of iterative refinements through the AlphaFold2 architecture.

Reasoning that the different variants might be recruiting different sequences from MMseqs2 for constructing the MSA that is utilized by ColabFold to determine the interacting residue pairs in the three‐dimensional structure, we ran ColabFold again, but using the same MSA derived for WT RfaH for all mutants, using 3 recycles (Figures S11–S14) and 12 recycles (Figures S16–S19) with and without dropouts. When using 3 recycles without dropouts, six out of the eight RfaH variants (F126A, E136A, R138A, S139A, L142A, and I146A) had a similar or higher number of predicted structures with a mixed α/β‐folded CTD in comparison to E48A (Figure S15). When dropouts are enabled, variants F126A, E136A, and S139A show a slight decrease in the number of predicted structures with mixed α/β‐folded CTD, whereas R138A, L142A, and L143A show an increase in the number of predicted structures with mixed secondary structure (Figure S15).

Repeating the same exercise with 12 recycles (Figures S16–S19) shows a similar trend, with F126A, E136A, R138A, L142A, and L143 showing a similar or higher number of models with mixed secondary structure when compared to E48A (Figure S20), with only a slight decrease in the number of models with such secondary structure content when compared to the ColabFold predictions using MSAs generated for each RfaH variant (Figure 3a), and with RfaH I129A now always predicted in the all‐α CTD conformation (i.e., all structures were grouped in one cluster).

As observed from some of the representative structures from this *k*‐means clustering, several structures exhibit rotations of the CTD with respect to the orientation of the NTD (e.g., representative structure of RfaH 138A in cluster 0, Figure S3). Additionally, a higher RMSD can imply the presence of β‐folded secondary structure or the absence of secondary structure for this region. However, the use of RMSD as a metric for comparison of the full‐length structures of RfaH variants is too sensitive to changes in flexible regions, such as the loop comprising residues 101–113 that connect the NTD and CTD. Therefore, once we established that the use of a higher number of recycles was a better approach for determining with residues might be relevant for the fold‐switch of RfaH, we implemented a hierarchical clustering approach based on the use of TM‐align (Zhang & Skolnick, 2005) to calculate the TM‐score, which is a better metric to evaluate the global topology between different structures, for all structures predicted using 12 recycles. In this approach, for each RfaH variant, we first determined the TM‐score of all structures against the best predicted structure for that given variant (Rank 1) and used a dissimilarity metric (e.g., 1—TM‐score) to generate a distance matrix for hierarchical clustering. By constructing dendrograms based on this distance matrix (Figures S21–S28), we were able to define a dissimilarity threshold of 0.12 to determine the number of clusters for each RfaH variant.

Using this approach, we were able to observe the broader diversity of topological configurations of the ColabFold‐predicted structures for each RfaH variant. For example, in the absence of dropouts, RfaH variants R138A and I146A explore conformations in which residues 114–131 are unfolded. For several alanine scanning variants, a topology in which the CTD is rotated ~45° with respect to its experimentally solved conformation is also observed (Figure S22). Interestingly, upon enabling dropouts, the number of clusters for many of these alanine scanning variants is significantly reduced (Figure S24). In comparison, when using the same MSA as the one for WT RfaH for all structure predictions, this trend is not observed (Figures S26 and S28).

Given the success of this approach in better resolving these diverse global topologies, we again counted the number of structures with mixed α/β content in the CTD. As can be seen in Figure 3b, the results are consistent with the analysis derived from *k*‐means clustering, indicating that F126A, E136A, R138A, and L142A variants have a similar or higher number of models with the two‐stranded antiparallel β‐sheet for residues 114–131 when compared to E48A. Moreover, these results do not significantly change when using the same MSA as in WT RfaH for all structure predictions of all RfaH variants.

Altogether, these structure predictions suggest that alanine substitutions F126A, E136A, R138A, and L142A might have a stronger effect on breaking the ID interactions that stabilize the autoinhibited state of RfaH.

### 2.3 In vivo assays validate the role of these CTD residues in stabilizing the autoinhibited state of RfaH

Our all‐atom SBM‐based MD simulations support the idea that alanine substitutions of I129, E136, R138, S139, L142, L143, I146, and N147 CTD residues destabilize the autoinhibited state of RfaH, due to the participation of these residues in ID interactions that persist with a high probability even in the transition state for NTD–CTD dissociation. Additionally, AlphaFold2‐based protein structure predictions highlight that variants F126A, E136A, R138A, and L142A lead to the prediction of mixed α/β CTD for the full‐length RfaH sequence. Based on this information, we designed an in vivo experimental assay to ascertain the role of some of these residues in RfaH fold‐switch.

Our in vivo experimental assays rely on the coupling between transcription and translation, with RfaH acting as a physical tether between the RNAP and the ribosome through CTD binding to the ribosomal protein S10 (Burmann et al., 2012). Thus, we examined recent cryoEM structures of the RfaH‐tethered transcription‐translation complexes (Molodtsov et al., 2024) to determine if these alanine scanning mutants could have detrimental effects on S10 binding and, by extension, on our activity assays. Indeed, residues F126, L143, I146, and N147 directly interact with S10 (Figure 3d), and (except for residue F126) will be occluded based on computational analysis of solvent‐accessible surface area (Table S3) (Fraczkiewicz & Braun, 1998). Thus, we excluded these variants from further experimental analysis. We also ignored the R138A variant, as its role in salt‐bridging with E48A to stabilize ID interactions is already well known (Burmann et al., 2012).

Given the aforementioned results, we selected five CTD residues for alanine scanning and subsequent experimental testing using in vivo gene expression assays of RfaH activity: E136 and L142, which were determined by both SBM‐based MD simulations and AlphaFold2 to be important for the stability of the ID interactions that regulate the fold‐switch of RfaH (Figures 2 and 3); I129 and S139, which were only ascertained by our MD simulations to be important for the stability of the ID interactions (Figure 2); and V154, a residue with low contact probability and no significant contribution to heterogeneous structure predictions using AlphaFold2, thus expected to have no effect on NTD‐CTD dissociation (Figures 2 and 3). We also used the E48A variant as a control for RfaH activation due to disruption of ID interactions (Burmann et al., 2012).

Based on our in vivo luciferase (*lux*) reporter assay developed for RfaH (Belogurov et al., 2010), two plasmids were co‐transformed into *E. coli* DH5α Δ*rfaH* cells: one encoding a selected RfaH variant and another with the *ops* element upstream of the *luxCDABE* operon. For the second plasmid, two versions of the *ops* element were used: a WT *ops* (*ops* ^*WT*^) and a mutant *ops* (*ops* ^*G8C*^); the latter still induces RNAP pausing at the *ops* site but abolishes RfaH recruitment (Zuber et al., 2018). The *lux* reporter plasmids lack the ribosome binding site (RBS), making the *lux* expression completely dependent on RfaH activity (Figure S30).

**FIGURE 4:**
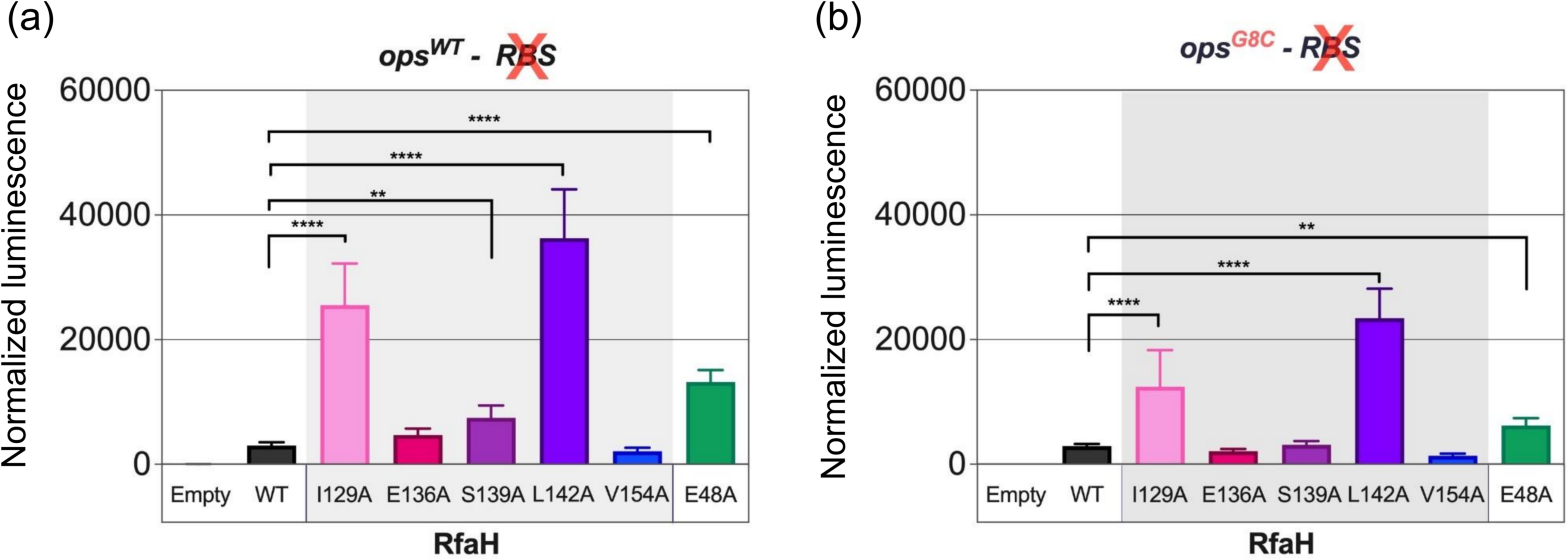
In vivo activity of alanine scanning substitutions of RfaH CTD. *Escherichia coli* DH5α *ΔrfaH* strains were co‐transformed with a plasmid containing indicated RfaH variants and another with different *ops* variants upstream of the *luxCDABE* operon: *ops* ^*WT*^ (a) and *ops* ^*G8C*^ (b). Both *ops*‐*luxCDABE* plasmids lacked the RBS site. Luminescence was normalized by cell density. The graph shows the mean value and the standard deviation of the measurements, which were made with six technical replicates of three different colonies. Statistical significance was determined using one‐way analysis of variance (ANOVA); (**p < 0.005; ****p < 0.0001).

If a given alanine substitution occurred in a residue crucial for the stability of the autoinhibited state of RfaH, that RfaH variant would be expected to have a higher propensity to fold‐switch into the active state and thus show higher activity than WT RfaH. This is indeed what we observed for RfaH I129A and L142A, which exhibit higher luminescence than WT RfaH, even surpassing the observed luminescence for RfaH E48A that was used as a positive control in both versions of the *ops* element (Figure 4a,b).

Among other substitutions, only S139A showed a statistically significantly higher luminescence than WT RfaH for *ops* ^WT^, but lower luminescence than the E48A variant (Figure 4a). In contrast, the E136A substitution did not show higher luminescence in either *ops* element context (Figure 4a,b). Lastly, RfaH V154 did not lead to the activation of RfaH, as expected based on the computational results.

Overall, these results are in good agreement with the computational information derived either from SBM‐based MD simulations and/or from protein structure predictions using AlphaFold2.

## 3 DISCUSSION

In this work, we presented a combination of all‐atom SBM‐based MD simulations, ColabFold protein structure predictions, and in vivo activity assays to ascertain which CTD residues are important for the fold‐switch of the metamorphic protein RfaH, beyond the already identified residues I93 and F130 (Shi et al., 2017).

No direct correlation was found between the ID contact probabilities derived from our MD simulations and the subsequent protein structure predictions. Of the RfaH variants tested in this work, MD simulations suggested that S139 might have a major contribution to the autoinhibited state of RfaH due to its high ID contact probability, whereas AlphaFold2 suggested that L142 might contribute to the stability of this state due to the higher number of predicted structures with mixed secondary structure of its alanine mutant.

The inconsistencies between SBMs and ColabFold predictions might arise from the significant differences between these two methods. SBMs encode contacts based on an input structure, which in this case, correspond to the autoinhibited state of RfaH solved by x‐ray crystallography. In contrast, AlphaFold2 infers contacts based on evolutionary information harnessed from thousands of homologous sequences, such that the residue pairs in contact determined by the EvoFormer module are not necessarily fully consistent with the native contacts encoded by the SBM model used herein.

Despite these inconsistencies between SBMs and ColabFold, in vivo experiments allowed us to confirm that I129 and L142 make an important contribution to the stabilization of the autoinhibited state by participating in interactions with the NTD.

It is worth noting that residues I129 and L142 are located in the region of the α‐helical hairpin, residues 125–145, which have been suggested to constitute the most stable CTD region in full‐length autoinhibited RfaH due to its interactions with the NTD by both simulations of the fold‐switch of full‐length RfaH (González‐Higueras et al., 2024; Ramírez‐Sarmiento et al., 2015) and experiments on the full‐length protein either in isolation (Cai et al., 2024; Galaz‐Davison et al., 2020) or interacting with RNAP (Molina et al., 2022; Zuber et al., 2024).

Among these residues, L142 was previously identified as a potentially important residue for the NTD‐CTD interaction due to its large and hydrophobic contact area, thus potentially participating in the stabilization of the autoinhibited state of RfaH (Shi et al., 2017). Similarly, a combination of local energetic frustration and evolution also identified L142 as a relevant residue for the fold‐switch of RfaH (Freiberger et al., 2023). Nevertheless, the importance of this residue for controlling the fold‐switch of RfaH has not been experimentally tested until this work.

Other CTD residues previously suggested to stabilize the autoinhibited state based on the ID interface contact area are I129, E136, and S139 (Shi et al., 2017). Among them, our experiments demonstrate that only I129 makes an important contribution to the stabilization of the autoinhibited state, but to a lesser extent than L142. In contrast, the higher activity of RfaH S139A relative to WT RfaH suggests a slight destabilization, which is not sufficient for activation in the absence of the *ops* signal, indicating that the structural transformation signal is still required for this variant to switch from the autoinhibited to the active state. The position of I129 next to F130, a residue with a dual role in RfaH fold‐switching (Ramírez‐Sarmiento et al., 2015), might indicate a possible cooperation between these two residues for the stabilization of the ID interaction. Lastly, E136A did not show any significantly higher activity than WT RfaH. Similar to WT RfaH, in the absence of RBS, pausing at the *ops* site expected to promote the recruitment of 30S is not enough to endorse the interaction between ribosome and RfaH E136A (Zuber et al., 2018).

It is possible that RfaH variants that were computationally identified to be relevant for the stability of the autoinhibited state exhibited low activity as a result of low protein expression or stability. This possibility was assessed in a previous study of single‐point mutations of RfaH NTD using Western blotting with a polyclonal antibody directed against the CTD (Belogurov et al., 2010). However, in the current study, residue substitutions in the CTD could affect antibody recognition, precluding the accurate assessment of the variants' expression levels.

Further understanding of the role of RfaH residues in encoding a fold‐switch may be gained from comparing their identity and conservation with the non‐metamorphic NusG paralog. A MSA made with hundreds of RfaH and thousands of NusG sequences revealed that I129, E136, R138, L142, and L143 are highly conserved across RfaH sequences (Shi et al., 2017). RfaH residues I129 and L142 are replaced in NusG by loosely conserved residues V147 and S161, whereas L143 is replaced by a highly conserved V162, and E136 and R138 are conserved across RfaH and NusG (Shi et al., 2017). Thus, the residues identified in this work as relevant for the fold‐switch of RfaH using MD simulations and protein structure predictions are consistent with their high conservation during the evolution of RfaH sequences.

In conclusion, we hypothesize that the combination of computationally efficient SBM‐based MD simulations to infer residues with high contact probability in the transition state ensemble of metamorphic proteins, combined with in silico alanine scanning mutagenesis using AlphaFold2, can guide efforts in determining which residues are important in controlling the fold‐switch of other metamorphic proteins for further experimental validation.

## 4 MATERIALS AND METHODS

### 4.1 Preparation of the structure of the autoinhibited state of RfaH

A complete structure for full‐length RfaH in the autoinhibited state was generated as in previous works (González‐Higueras et al., 2024), using the solved structures of full‐length RfaH: Protein Data Bank (PDB) identification code 5OND, chain B, lacking residues 99–118 and 154–162; and PDB 2OUG, chain B, lacking residues 101–114 and 157–162. The NTD (residues 1–99) was taken from PDB 5OND, and the CTD (residues 115–156) was taken from the merge of residues 121–151 from PDB 5OND with residues 115–120 and 152–156 from PDB 2OUG after structural superimposition. All missing residues in the full‐length protein (residues 100–114 and 157–162) were added and refined using the automodel class of MODELLER v9.23 (Webb & Sali, 2016) while keeping all other experimental atom positions fixed.

This model was further subjected to minimization in explicit solvent using GROMACS v4.5.4 (Hess et al., 2008) and the Amber ff99SB‐ILDN force field (Lindorff‐Larsen et al., 2010). Briefly, each system was solvated in a cubic box of 1.0 nm of padding with TIP3P water molecules, neutralized with counterions, and minimized using the steepest descent method.

### 4.2 Generation of all‐atom single‐basin structure‐based models

The energy‐minimized structure of the autoinhibited state of RfaH was used for generating a single‐basin all‐atom SBM (Whitford et al., 2009) with Gaussian contact potentials (Noel et al., 2012) using SMOG2 v2.2 (Noel et al., 2016). This model explicitly represents all heavy atoms in the protein as beads of unit mass, whereas Gaussian potentials enable setting the excluded volume for each native contact independently of the contact minima (Retamal‐Farfán et al., 2023).

The functional form of the potential, which is described in detail elsewhere (Whitford et al., 2009), is:

(1) $$V_{\mathrm{sb}}=\begin{array}{l} \sum_{\text{bonds}}\varepsilon_{r}{\left(r-r_{0}\right)}^{2}+\sum_{\text{angles}}\varepsilon_{\theta }{\left(\theta -\theta_{0}\right)}^{2}\\ +\;\sum_{\text{improper}/\text{planar}}\varepsilon_{\chi }{\left(\chi -\chi_{0}\right)}^{2}+\sum_{\text{backbone}}\varepsilon_{\mathrm{BB}}F_{D}\left(\varphi -\varphi_{0}\right)\\ +\;\sum_{\text{sidechains}}\varepsilon_{\mathrm{SC}}F_{D}\left(\varphi -\varphi_{0}\right)+\sum_{\text{contacts}}\varepsilon_{C}C\left(r_{\mathrm{ij}},r_{0}^{\mathrm{ij}}\right)\\ +\;\sum_{\text{non}-\text{contacts}}\varepsilon_{\mathrm{NC}}{\left(\frac{\sigma_{\mathrm{NC}}}{r_{\mathrm{ij}}}\right)}^{12}, \end{array}$$

where $r_{0}$, $\theta_{0}$, $\chi_{0}$, $\varphi_{0}$, and $r_{0}^{\mathrm{ij}}$ denote the value of bonds, angles, dihedrals and atom‐atom contact distances in the native state; and $\varepsilon_{r}$, $\varepsilon_{\theta }$, $\varepsilon_{\chi }$, $\varepsilon_{\mathrm{BB}}$, $\varepsilon_{\mathrm{SC}}$, $\varepsilon_{C}$, and $\varepsilon_{\mathrm{NC}}$ denote the interaction strengths for bonds, angles, planar dihedrals, backbone dihedrals, sidechain dihedrals, atom‐atom contacts and non‐native atom‐atom repulsions. Non‐native atom‐atom repulsions are defined at a distance *σ*_NC_ = 2.5 Å, whereas dihedrals are treated using a traditional dihedral potential:

(2) $$F_{D}=\left[1-\cos\left(\varphi -\varphi_{0}\right)\right]+\frac{1}{2}\left[1-\cos\left(3\left(\varphi -\varphi_{0}\right)\right)\right].$$

In these all‐atom SBMs, reduced energy units are used, such that *ɛ*_r_ = 10,000ε; *ɛ*_θ_ = 80ε; $\varepsilon_{\chi }=10\varepsilon$; $\varepsilon_{\mathrm{NC}}$ = 0.1ε, where ε is the reduced energy unit. The values of $\varepsilon_{\mathrm{BB}}$, $\varepsilon_{\mathrm{SC}}$, and $\varepsilon_{C}$ are assigned such that $\varepsilon_{\mathrm{BB}}$ and $\varepsilon_{\mathrm{SC}}$ are scaled as $\varepsilon_{\mathrm{BB}}/\varepsilon_{\mathrm{SC}}$ = 2, the ratio of total contact energy to total dihedral energy is scaled as $\sum \varepsilon_{c}/\left(\sum \varepsilon_{\mathrm{BB}}+\sum \varepsilon_{\mathrm{SC}}\right)=2$ and the total stabilizing energy $\sum \varepsilon_{C}+\sum \varepsilon_{\mathrm{BB}}+\sum \varepsilon_{\mathrm{SC}}=\varepsilon N_{\text{atoms}}$.

Non‐bonded atom pairs that are in contact in the native state, that is, atom pairs between residues *i* and *j* with a sequence separation *i* > *j* + 3, within a cutoff distance of 6 Å and further filtered by a “shadow” screening parameter that discards occluded contacts (Noel et al., 2012), are given single‐basin Gaussian well contact potentials:

(3) $$C_{G}\left(r_{\mathrm{ij}},r_{0}^{\mathrm{ij}}\right)=\left(1+\varepsilon_{\mathrm{NC}}{\left(\frac{\sigma_{\mathrm{NC}}}{r_{\mathrm{ij}}}\right)}^{12}\right)\left(1+G\left(r_{\mathrm{ij}},r_{0}^{\mathrm{ij}}\right)\right)-1.$$

(4) $$G\left(r_{\mathrm{ij}},r_{0}^{\mathrm{ij}}\right)=-\exp\left[\frac{-{\left(r_{\mathrm{ij}}-r_{0}^{\mathrm{ij}}\right)}^{2}}{\left(2\sigma^{2}\right)}\right];\sigma =\frac{0.2r_{0}^{\mathrm{ij}}}{\sqrt{2\ln2}}$$

All native contacts formed between the MODELLER‐added linker of RfaH and the rest of the protein were eliminated in the final SBM model.

### 4.3 All‐atom SBM‐based MD simulations

MD simulations using our all‐atom SBM for RfaH were performed using a modified version of GROMACS v4.5.4 with Gaussian potentials (Noel et al., 2012). The simulations were run for 2.5 × 10^8^ integration steps at 31 different temperatures ranging from 1.04 to 1.33 reduced units, using a timestep of 0.002 reduced units. The explored protein configurations and corresponding energies during these simulations were stored every 1000 steps.

Then, the extension g_kuh, available in the modified version of GROMACS v4.5.4 (Noel et al., 2012), was employed to calculate the fraction of residue pair native contacts formed for each sampled configuration with respect to the total number of native contacts (Q), the native contacts in the RfaH NTD (Q_NTD_), CTD (Q_CTD_), and the ID native contacts between the NTD and CTD (Q_ID_). These values were used as reaction coordinates, along with the potential energy of each configuration, to calculate the heat capacity and free energy profiles of RfaH using WHAM (Kumar et al., 1992) implemented as a Java application in SMOG2 (Noel et al., 2016).

### 4.4 In silico alanine scanning mutagenesis and AlphaFold2 protein structure prediction

RfaH CTD residues with high contact probability in the transition state ensemble for NTD‐CTD dissociation (I129, E136, R138, S139, L142, L143, I146, and N147), as well as two randomly selected residues with low contact probability (F126, V154) and the known E48 residue that participates in salt‐bridging the NTD and CTD via interactions with R138, were subjected to in silico alanine scanning mutagenesis, and the resulting full‐length RfaH sequences were used as input for protein structure prediction using ColabFold (Mirdita et al., 2022), a cloud‐computing version of AlphaFold2 (Jumper et al., 2021).

Several parameters were modified in ColabFold to ensure appropriate sampling for all RfaH variants. We utilized all five model parameters of AlphaFold2, while increasing the number of random seeds from 1 to 10, such that a total of 50 predicted structures were generated per RfaH variant per condition. We used either 3 recycles or 12 recycles of iterative refinement, and performed structure predictions without dropouts or with dropouts enabled, which are known to increase the structural diversity of the sampled models (Wallner, 2023). Lastly, since ColabFold retrieves homologous sequences for generating the MSA that is used by the EvoFormer using MMseqs2 (Steinegger & Söding, 2017), which could lead to differences in the sequences retrieved at this stage for different RfaH variants, we repeated all structure predictions using the same MSA that was retrieved from MMseqs2 for WT RfaH. Thus, a total of 4800 structures (400 structures for each of the 12 RfaH variants tested) were predicted using ColabFold under all these conditions.

### 4.5 Analysis of ColabFold‐predicted structures

Given the large number of structures to analyze for determining the effects of site‐specific alanine scanning mutations, two clustering approaches were utilized to discriminate between structures whose CTD is folded as an α‐helical hairpin or whether it shows some β‐strand secondary structure content.

In the first approach, we utilized PyTraj (Roe & Cheatham III, 2013) to superimpose and calculate the RMSD of the Cα of CTD residues 126–131 for all 50 structures, generated using ColabFold using a given set of parameters, against the best predicted structure of the set (Rank 1). It is worth noting that, in all cases and sets of parameters, the CTD of the predicted structure ranked as 1 is folded as an α‐helical hairpin. Then, we used *k*‐means clustering based on these RMSD values, identifying the optimal number of clusters *k* using the elbow method, with the exception that if the maximum observed RMSD across all 50 structures was <1.2 Å, the structures were clustered into a single group. The results of this clustering analysis and representative structures of these clusters can be found in Figures S2–S5 (parameters: 3 recycles, 10 seeds, five model parameters, with and without dropouts), Figures S7–S10 (parameters: 12 recycles, 10 seeds, five model parameters, with and without dropouts), Figures S11–S14 (parameters: 3 recycles, 10 seeds, five model parameters, with and without dropouts, same MSA as WT RfaH), and Figures S16–S19 (parameters: 12 recycles, 10 seeds, five model parameters, with and without dropouts, same MSA as WT RfaH).

Given that this local measurement does not take into account the global fold of the predicted structure, such as the orientation of the CTD with respect to the NTD, and that a high RMSD can also imply an unfolded structure rather than a β‐strand, we performed a second clustering analysis based on the TM‐score, a metric for assessing the topological similarity of protein structures, calculated against the best predicted structure (Rank 1) using TM‐align (Zhang & Skolnick, 2005). The TM‐score values against the best predicted structure were used to generate a dissimilarity score (i.e., 1—TM‐score), which was used to generate a distance matrix for hierarchical clustering. All 50 structures generated for each variant under different sets of ColabFold parameters were assigned to different clusters based on a dissimilarity score threshold of 0.12. This analysis was performed only for the predicted structures using 12 recycles of iterative refinement, and the results of the clustering analysis and representative structures of these clusters can be found in Figures S21–S24 (parameters: 12 recycles, 10 seeds, five model parameters, with and without dropouts), and Figures S25–S28 (parameters: 12 recycles, 10 seeds, five model parameters, with and without dropouts, same MSA as WT RfaH).

### 4.6 Plasmids and strains

The list of plasmids used in this work can be found in Table S1. RfaH with alanine substitutions was constructed through site‐directed mutagenesis, with primers listed in Table S2. An *E. coli* DH5α Δ*rfaH* (IA149) strain (Belogurov et al., 2010) was used for the co‐transformation of plasmids necessary for RfaH‐dependent *lux* reporter assays.

### 4.7 In vivo RfaH luminescence reporter assays

Plasmids encoding RfaH variants were co‐transformed with vectors containing the *lux* reporter vector into the IA149 strain. The transformed cells were plated in Luria‐Bertani (LB) media supplemented with carbenicillin (100 μg/mL) and chloramphenicol (30 μg/mL). The single colonies were inoculated in 5 mL LB media with the same concentration of antibiotics and incubated overnight at 37°C with agitation at 250 rpm. The next day, the overnight cultures were diluted 1:100 into fresh 3‐(N‐morpholino)propanesulfonic acid EZ Rich Defined Media (M2105, Teknova) containing antibiotics and 0.2% glucose and grown for 6 h at 37°C with agitation. RfaH variants were induced with 0.2 mM of isopropyl β‐D‐thiogalactopyranoside for an additional 1.5 h at 37°C with agitation. Luminescence was measured in 200 μL using six technical replicates and three biological replicates on a FLUOstar OPTIMA plate reader (BMG LABTECH GmbH, Offenburg, Germany) and normalized by cell density. The data was analyzed in Microsoft Excel.

## AUTHOR CONTRIBUTIONS

**Cyndi Tabilo‐Agurto:** Conceptualization; data curation; formal analysis; funding acquisition; investigation; methodology; visualization; writing – original draft; writing – review and editing. **Javiera Reyes:** Conceptualization; data curation; formal analysis; funding acquisition; investigation; methodology; visualization; writing – original draft; writing – review and editing. **Irina Artsimovitch:** Conceptualization; methodology; data curation; funding acquisition; writing – original draft; formal analysis; investigation; writing – review and editing. **César A. Ramírez‐Sarmiento:** Conceptualization; data curation; formal analysis; investigation; funding acquisition; methodology; resources; visualization; writing – original draft; writing – review and editing.

## CONFLICT OF INTEREST STATEMENT

The authors declare no conflict of interest exists.

## Supporting information


**Data S1.** Tables S1–S3 and Figures S1–S30 are available in the Supporting Information S1. SBM models for RfaH, the predicted structures of all RfaH variants using ColabFold with different parameters (number of recycles, number of seeds, use of dropouts, use of a single MSA) and Jupyter Notebooks for clustering analysis of the resulting structures using RMSD and TM‐score, are available at Zenodo (https://doi.org/10.5281/zenodo.15265404).

## Data Availability

The data that support the findings of this study are openly available in Zenodo at https://doi.org/10.5281/zenodo.15265404.
