# Supplementary material for: Computational and experimental assessment of key interdomain residues controlling the fold‐switch of RfaH
Source: Protein Sci. 2025 Jun 16;34(7):e70202. doi: 10.1002/pro.70202 (PMC12168481; doi:10.1002/pro.70202)
Supplement: Supplementary file 1 — Data S1. Tables S1–S3 and Figures S1–S30 are available in the Supporting Information S1. SBM models for RfaH, the predicted structures of all RfaH variants using ColabFold with different parameters (number of recycles, number of seeds, use of dropouts, use of a single MSA) and Jupyter Notebooks for clustering analysis of the resulting structures using RMSD and TM‐score, are available at Zenodo (https://doi.org/10.5281/zenodo.15265404). [file PRO-34-e70202-s001.pdf]

SUPPORTING INFORMATION

Computational and experimental assessment of key interdomain residues controlling the fold-switch of RfaH

Cyndi Tabilo-Agurto[1,2], Javiera Reyes[1,2], Irina Artsimovitch[3,*], César A. Ramírez-Sarmiento[1,2,*]

[1]Institute for Biological and Medical Engineering, Schools of Engineering, Medicine and Biological Sciences, Pontificia Universidad Católica de Chile, Santiago 7820436, Chile

[2]ANID, Millennium Science Initiative Program, Millennium Institute for Integrative Biology (iBio), Santiago 833150, Chile

[3]Department of Microbiology and Center for RNA Biology, The Ohio State University, Columbus, OH 43210, USA

*Correspondence should be addressed to:

Irina Artsimovitch, Department of Microbiology and The Center for RNA Biology, The Ohio State University, 484 W. 12th Ave, Columbus, OH 43210, USA; +1 614 688 3561; artsimovitch.1@osu.edu

César A. Ramírez-Sarmiento, Institute for Biological and Medical Engineering, Schools of Engineering, Medicine and Biological Sciences, Pontificia Universidad Católica de Chile, Av. Vicuña Mackenna 4860, Santiago 7820436, Chile; +56 2 2354 1110; cesar.ramirez@uc.cl

This Supporting Information contains:

- Tables S1-S3
- Figures S1-S30

Table S1. Plasmids Information

Plasmids	Name	Description	Resistance	Reference
RfaH expression vectors	pIA947	P_{trc} – empty	Cm	26
	pIA957	P_{trc} – *RfaH*^{WT}<br>RfaH inserted between *Nde*I and *Hin*dIII in pIA947	Cm	26
	pJR12	P_{trc} – *RfaH*^{I129A}<br>RfaH inserted between *Nde*I and *Hin*dIII in pIA947	Cm	This work
	pJR13	P_{trc} – *RfaH*^{E136A}<br>RfaH inserted between *Nde*I and *Hin*dIII in pIA947	Cm	This work
	pJR14	P_{trc} – *RfaH*^{S139A}<br>RfaH inserted between *Nde*I and *Hin*dIII in pIA947	Cm	This work
	pJR15	P_{trc} – *RfaH*^{L142A}<br>RfaH inserted between *Nde*I and *Hin*dIII in pIA947	Cm	This work
	pJR16	P_{trc} – *RfaH*^{V154A}<br>RfaH inserted between *Nde*I and *Hin*dIII in pIA947	Cm	This work
luxCDABE reporter vectors	pIA1087	P_{BAD} – *ops*^{WT} – *luxCDABE* without RBS	Amp	8
	pZL23	P_{BAD} – *ops*^{G8C} – *luxCDABE* without RBS	Amp	32

Table S2. Primers for site-directed PCR mutagenesis

RfaH Variant	Forward/Reverse	Primer Sequence
I129A	Forward	5’-GAA GGC TTT CAG GCC GCG TTC ACC GAA C-3’
I129A	Reverse	5’-GGC CTG AAA GCC TTC GAA CGC GCC T-3’
E136A	Forward	5’-ACC GAA CCC GAT GGT GCG GCT CGC TCC A-3’
E136A	Reverse	5’-ACC ATC GGG TTC GGT GAA AAT GGC CTG A-3’
S139A	Forward	5’-GAT GGT GAG GCT CGC GCG ATG CTA TTG C-3’
S139A	Reverse	5’-GCG AGC CTC ACC ATC GGG TTC GGT G-3’
L142A	Forward	5’-GCT CGC TCC ATG CTA GCG CTT AAT CTT A-3’
L142A	Reverse	5’-TAG CAT GGA GCG AGC CTC ACC ATC G-3’
V154A	Forward	5’-AGA TTA AGC ACA GTG CGA AGA ATA CC-3’
V154A	Reverse	5’-CAC TGT GCT TAA TCT CTT TAT TA-3’

Table S3. Solvent accessible surface area from GetArea for RfaH (chain AB) and S10 (chain P) from the cryoEM structure of RfaH-tethered transcription-translation complex (PDB 8UQP)

**Probe radius**		**1.4 Å**					
**Chain: P**		**Ribosomal Protein S10**					
Res. Name	Res. Number	Total	Apolar	Backbone	Sidechain	Ratio(%)	In/Out
ARG	5	125.30	40.05	17.83	107.46	55.0	o
ILE	6	2.87	2.87	0.00	2.87	1.9	i
ARG	7	69.79	26.95	0.00	69.79	35.7	
ILE	8	2.47	2.47	0.00	2.47	1.7	i
ARG	9	81.85	38.36	0.42	81.43	41.7	
LEU	10	0.07	0.05	0.02	0.05	0.0	i
LYS	11	35.12	34.19	0.94	34.19	20.8	
ALA	12	5.90	5.90	5.90	0.00	0.0	i
PHE	13	132.06	121.53	10.53	121.53	67.5	o
ASP	14	68.00	28.45	6.51	61.49	54.4	o
HIS	15	102.67	78.62	0.00	102.67	66.4	o
ARG	16	181.90	87.35	1.70	180.20	92.2	o
LEU	17	50.54	50.54	2.95	47.58	32.5	
ILE	18	0.00	0.00	0.00	0.00	0.0	i
ASP	19	60.29	24.79	2.66	57.63	51.0	o
GLN	20	109.89	36.51	3.99	105.90	73.7	o
ALA	21	0.58	0.58	0.57	0.00	0.0	i
THR	22	9.57	0.70	0.00	9.57	9.0	i
ALA	23	44.56	44.56	7.24	37.32	57.5	o
GLU	24	45.00	38.11	4.67	40.33	28.6	
ILE	25	3.10	3.10	0.00	3.10	2.1	i
VAL	26	21.03	21.03	0.00	21.03	17.2	i
GLU	27	71.84	52.31	0.11	71.73	50.8	o
THR	28	9.93	9.93	0.01	9.92	9.3	i

ALA	29	1.34	1.14	0.20	1.14	1.8	i
LYS	30	97.98	84.07	12.93	85.05	51.7	o
ARG	31	163.04	93.64	34.11	128.93	65.9	o
THR	32	38.42	24.59	18.98	19.44	18.3	i
GLY	33	13.18	4.19	13.18	0.00	15.1	i
ALA	34	54.49	53.09	7.79	46.69	71.9	o
GLN	35	118.94	64.31	18.78	100.16	69.7	o
VAL	36	29.01	9.30	19.72	9.30	7.6	i
ARG	37	143.39	42.16	17.99	125.40	64.1	o
GLY	38	13.24	13.24	13.24	0.00	15.2	i
PRO	39	75.41	64.89	10.53	64.88	61.7	o
ILE	40	78.42	78.42	1.57	76.85	52.2	o
PRO	41	97.49	81.18	16.30	81.18	77.2	o
LEU	42	47.16	39.22	8.61	38.55	26.4	
PRO	43	117.36	115.49	14.52	102.84	97.8	o
THR	44	45.83	10.45	19.74	26.09	24.6	
ARG	45	158.03	74.72	6.23	151.80	77.6	o
LYS	46	92.51	44.01	16.35	76.15	46.3	
GLU	47	27.06	25.73	9.02	18.03	12.8	i
ARG	48	147.27	55.41	20.25	127.02	65.0	o
PHE	49	111.78	111.78	9.44	102.33	56.8	o
THR	50	70.22	32.43	21.14	49.08	46.2	
VAL	51	67.73	67.73	6.07	61.66	50.4	o
LEU	52	86.39	68.99	17.41	68.99	47.2	
ILE	53	104.46	82.17	30.08	74.38	50.5	o
SER	54	26.08	9.87	15.59	10.49	13.5	i
PRO	55	148.49	119.29	43.58	104.91	99.7	o
HIS	56	160.42	114.82	22.61	137.81	89.1	o
VAL	57	101.23	90.10	14.42	86.80	71.0	o

ASN	58	88.67	16.85	5.97	82.71	72.4	o
LYS	59	111.99	67.44	4.42	107.56	65.4	o
ASP	60	140.68	47.37	24.43	116.26	100.0	o
ALA	61	35.35	35.00	12.26	23.09	35.6	
ARG	62	105.77	31.50	25.81	79.96	40.9	
ASP	63	42.46	18.90	7.37	35.09	31.1	
GLN	64	91.17	58.21	22.90	68.27	47.5	
TYR	65	126.10	97.21	5.65	120.45	62.4	o
GLU	66	74.77	30.41	23.16	51.61	36.6	
ILE	67	60.08	60.08	4.18	55.89	37.9	
ARG	68	111.36	30.10	2.53	108.84	55.7	o
THR	69	24.53	19.14	0.00	24.53	23.1	
HIS	70	26.31	24.33	0.13	26.18	16.9	i
LEU	71	31.78	31.34	0.43	31.34	21.4	
ARG	72	38.65	3.72	0.26	38.39	19.6	i
LEU	73	32.99	32.99	0.00	32.99	22.6	
VAL	74	0.01	0.01	0.00	0.01	0.0	i
ASP	75	3.96	0.00	0.00	3.96	3.5	i
ILE	76	8.17	8.17	0.00	8.17	5.5	i
VAL	77	61.92	53.11	8.81	53.11	43.4	
GLU	78	78.52	49.60	2.45	76.07	53.9	o
PRO	79	52.68	27.26	25.42	27.26	25.9	
THR	80	54.07	49.50	15.08	38.99	36.7	
GLU	81	96.70	45.53	17.10	79.60	56.4	o
LYS	82	117.19	85.71	1.19	116.00	70.5	o
THR	83	1.22	1.22	0.00	1.22	1.2	i
VAL	84	20.44	20.41	0.11	20.33	16.6	i
ASP	85	2.83	2.83	0.00	2.82	2.5	i
ALA	86	13.39	13.39	5.29	8.09	12.5	i

LEU	87	18.12	15.97	2.15	15.97	10.9	i
MET	88	44.75	25.32	20.00	24.76	15.6	i
ARG	89	144.06	81.17	31.64	112.42	57.5	o
LEU	90	50.34	49.08	8.51	41.83	28.6	
ASP	91	124.27	43.46	22.76	101.51	89.8	o
LEU	92	35.85	19.54	21.52	14.33	9.8	i
ALA	93	53.71	49.65	19.26	34.45	53.1	o
ALA	94	94.79	86.98	18.37	76.42	100.0	o
GLY	95	59.93	50.12	59.93	0.00	68.7	o
VAL	96	14.63	0.75	14.63	0.00	0.0	i
ASP	97	97.11	37.68	10.34	86.77	76.8	o
VAL	98	38.08	28.29	9.79	28.29	23.1	
GLN	99	93.10	29.10	5.25	87.85	61.1	o
ILE	100	10.15	5.09	5.06	5.09	3.5	i
SER	101	40.08	34.94	0.76	39.32	50.8	o
LEU	102	58.29	46.41	11.88	46.41	31.7	
GLY	103	69.97	48.64	69.97	0.00	80.2	o
**Chain: AB**		**Full-length RfaH (active state)**					
Res. Name	Res. Number	Total	Apolar	Backbone	Sidechain	Ratio(%)	In/Out
GLN	2	87.30	49.26	26.05	61.25	42.6	
SER	3	41.44	24.36	2.30	39.13	50.6	o
TRP	4	77.87	59.64	2.03	75.84	33.8	
TYR	5	29.56	10.65	0.00	29.56	15.3	i
LEU	6	33.50	33.42	0.07	33.42	22.9	
LEU	7	15.79	15.79	0.00	15.79	10.8	i
TYR	8	84.58	56.14	0.00	84.58	43.8	
CYS	9	0.26	0.19	0.06	0.20	0.2	i
LYS	10	106.53	60.15	9.34	97.19	59.1	o

ARG	11	151.04	92.19	11.31	139.73	71.5	o
GLY	12	78.28	44.87	78.28	0.00	89.8	o
GLN	13	94.96	37.12	9.85	85.11	59.2	o
LEU	14	67.98	67.74	7.68	60.30	41.2	
GLN	15	131.03	74.43	6.64	124.39	86.6	o
ARG	16	150.05	66.69	2.97	147.09	75.2	o
ALA	17	10.39	10.10	0.30	10.10	15.6	i
GLN	18	5.37	0.56	1.29	4.08	2.8	i
GLU	19	69.98	29.58	5.46	64.52	45.7	
HIS	20	71.91	47.24	11.85	60.07	38.9	
LEU	21	21.77	21.77	0.10	21.68	14.8	i
GLU	22	78.47	51.38	20.61	57.86	41.0	
ARG	23	145.42	86.03	39.44	105.98	54.2	o
GLN	24	85.54	47.35	20.52	65.02	45.2	
ALA	25	101.99	87.15	28.46	73.53	100.0	o
VAL	26	8.26	0.00	8.26	0.00	0.0	i
ASN	27	21.72	18.57	7.76	13.96	12.2	i
CYS	28	26.91	6.51	20.40	6.51	6.4	i
LEU	29	68.31	67.70	5.57	62.74	42.9	
ALA	30	12.04	7.37	6.72	5.31	8.2	i
PRO	31	20.10	20.10	0.09	20.02	19.0	i
MET	32	58.56	58.56	0.00	58.56	37.0	
ILE	33	10.85	10.85	0.00	10.85	7.4	i
THR	34	34.29	24.81	0.00	34.29	32.3	
LEU	35	23.92	23.91	0.01	23.91	16.4	i
GLU	36	26.45	25.40	0.05	26.40	18.7	i
LYS	37	40.47	40.37	0.23	40.25	24.5	
ILE	38	25.67	12.26	13.51	12.16	8.3	i
VAL	39	95.33	75.92	26.62	68.71	56.2	o

ARG	40	203.75	98.74	21.60	182.15	93.2	o
GLY	41	26.21	6.28	26.21	0.00	30.1	
LYS	42	151.37	107.25	6.98	144.39	87.8	o
ARG	43	117.19	67.94	23.11	94.08	48.1	
THR	44	56.08	49.74	5.64	50.44	47.5	
ALA	45	80.53	57.43	23.11	57.42	88.5	o
VAL	46	74.78	74.78	6.93	67.85	55.5	o
SER	47	70.31	22.60	19.17	51.14	66.1	o
GLU	48	70.86	14.72	2.73	68.13	48.3	
PRO	49	64.02	62.02	2.00	62.02	59.0	o
LEU	50	62.98	45.63	17.36	45.63	31.2	
PHE	51	81.70	68.32	13.39	68.32	37.9	
PRO	52	83.60	82.15	4.01	79.59	75.7	o
ASN	53	23.06	5.85	5.85	17.21	15.1	i
TYR	54	89.85	47.37	1.94	87.91	45.5	
LEU	55	21.79	21.79	0.94	20.85	14.3	i
PHE	56	16.76	16.76	0.00	16.76	9.3	i
VAL	57	6.57	6.57	0.00	6.57	5.4	i
GLU	58	25.55	1.81	0.00	25.55	18.1	i
PHE	59	30.23	30.07	0.16	30.07	16.7	i
ASP	60	46.02	7.27	0.02	46.01	40.7	
PRO	61	74.55	71.27	3.36	71.20	67.7	o
GLU	62	133.40	50.89	3.19	130.20	92.2	o
VAL	63	135.82	122.39	18.56	117.27	95.9	o
ILE	64	73.33	73.26	4.72	68.61	46.6	
HIS	65	126.36	100.73	3.23	123.12	79.6	o
THR	66	67.53	43.70	2.05	65.47	61.7	o
THR	67	115.09	79.29	11.23	103.86	97.8	o
THR	68	109.80	68.72	25.04	84.76	79.8	o

ILE	69	53.62	53.61	2.37	51.25	34.8	
ASN	70	74.98	12.40	6.92	68.06	59.5	o
ALA	71	109.47	80.98	45.26	64.21	98.9	o
THR	72	28.95	11.40	13.41	15.54	14.6	i
ARG	73	92.42	35.27	2.92	89.50	45.8	
GLY	74	11.35	8.62	11.35	0.00	13.0	i
VAL	75	72.43	56.71	16.74	55.69	45.5	
SER	76	51.95	17.66	27.15	24.79	32.0	
HIS	77	93.88	75.55	5.03	88.84	57.5	o
PHE	78	38.49	37.64	0.85	37.64	20.9	
VAL	79	35.65	35.65	5.86	29.80	24.4	
ARG	80	62.64	39.03	6.01	56.63	29.0	
PHE	81	211.23	182.42	44.72	166.51	92.5	o
GLY	82	49.13	44.03	49.13	0.00	56.3	o
ALA	83	106.58	90.82	30.35	76.24	100.0	o
SER	84	78.24	56.81	7.02	71.22	92.0	o
PRO	85	59.47	52.61	6.87	52.61	50.0	o
ALA	86	33.89	32.45	10.00	23.89	36.8	
ILE	87	134.20	117.30	16.90	117.30	79.6	o
VAL	88	14.52	5.28	10.36	4.16	3.4	i
PRO	89	109.55	105.81	13.24	96.31	91.5	o
SER	90	1.48	1.42	0.04	1.44	1.9	i
ALA	91	38.85	38.16	2.33	36.51	56.3	o
VAL	92	23.60	23.49	0.11	23.49	19.2	i
ILE	93	53.05	36.00	18.81	34.24	23.2	
HIS	94	128.66	81.35	37.59	91.08	58.9	o
GLN	95	138.76	41.05	33.61	105.15	73.2	o
LEU	96	102.08	102.02	11.38	90.70	62.0	o
SER	97	76.28	45.68	15.31	60.97	78.8	o

VAL	98	149.65	123.53	30.33	119.32	97.6	o
TYR	99	69.29	56.48	16.81	52.48	27.2	
LYS	100	57.29	26.59	10.68	46.61	28.3	
PRO	101	129.08	108.03	33.77	95.31	90.6	o
LYS	102	152.77	110.63	34.08	118.69	72.2	o
ASP	103	63.42	19.25	7.03	56.39	49.9	
ILE	104	78.67	78.67	0.00	78.67	53.4	o
VAL	105	65.05	64.56	0.87	64.18	52.5	o
ASP	106	0.18	0.00	0.02	0.16	0.1	i
PRO	107	92.98	85.53	8.62	84.36	80.2	o
ALA	108	102.31	84.16	38.75	63.56	97.9	o
THR	109	42.62	38.69	3.97	38.65	36.4	
PRO	110	35.58	34.90	4.86	30.73	29.2	
TYR	111	76.25	72.90	0.23	76.02	39.4	
PRO	112	85.78	69.79	15.99	69.79	66.3	o
GLY	113	32.40	20.80	32.40	0.00	37.2	
ASP	114	46.00	18.14	6.50	39.50	35.0	
LYS	115	113.93	80.25	15.10	98.83	60.1	o
VAL	116	33.93	33.01	2.53	31.40	25.7	
ILE	117	59.94	59.94	0.53	59.41	40.3	
ILE	118	5.74	5.70	0.04	5.70	3.9	i
THR	119	36.56	14.64	7.91	28.66	27.0	
GLU	120	89.09	29.08	0.09	89.00	63.0	o
GLY	121	41.31	30.21	41.31	0.00	47.4	
ALA	122	74.88	58.13	30.00	44.87	69.1	o
PHE	123	29.39	24.70	4.86	24.52	13.6	i
GLU	124	80.13	18.16	5.18	74.95	53.1	o
GLY	125	50.01	25.61	50.01	0.00	57.4	o
PHE	126	54.99	54.99	6.31	48.68	27.0	

GLN	127	127.92	33.45	18.65	109.27	76.0	o
ALA	128	3.45	3.32	0.40	3.05	4.7	i
ILE	129	21.71	21.71	0.00	21.71	14.7	i
PHE	130	3.95	3.95	0.00	3.95	2.2	i
THR	131	67.93	51.68	16.57	51.37	48.4	
GLU	132	54.88	36.47	0.00	54.88	38.9	
PRO	133	12.55	2.47	10.08	2.47	2.3	i
ASP	134	85.16	35.42	34.71	50.45	44.6	
GLY	135	44.39	42.03	44.39	0.00	50.9	o
GLU	136	23.73	16.10	5.06	18.67	13.2	i
ALA	137	34.74	34.55	0.19	34.55	53.2	o
ARG	138	134.08	59.42	15.38	118.71	60.7	o
SER	139	35.63	22.34	17.46	18.17	23.5	
MET	140	145.63	144.29	1.34	144.29	91.1	o
LEU	141	2.58	2.58	0.00	2.58	1.8	i
LEU	142	30.79	30.79	0.00	30.79	21.1	
LEU	143	17.55	17.55	1.16	16.39	11.2	i
ASN	144	97.28	31.07	6.73	90.54	79.2	o
LEU	145	20.24	7.66	13.25	6.98	4.8	i
ILE	146	84.02	58.68	34.97	49.05	33.3	
ASN	147	40.92	8.69	27.25	13.67	12.0	i
LYS	148	61.08	50.15	6.19	54.88	33.4	
GLU	149	109.22	68.09	16.94	92.28	65.4	o
ILE	150	47.14	47.14	10.95	36.19	24.6	
LYS	151	61.46	57.03	4.39	57.07	34.7	
HIS	152	0.34	0.34	0.00	0.34	0.2	i
SER	153	37.01	16.12	15.61	21.39	27.6	
VAL	154	0.64	0.64	0.64	0.00	0.0	i
LYS	155	96.70	64.99	0.00	96.70	58.8	o

ASN	156	53.06	13.96	0.00	53.06	46.4	
THR	157	72.84	62.04	0.66	72.19	68.0	o
GLU	158	50.20	6.89	0.00	50.20	35.6	
PHE	159	0.64	0.44	0.20	0.44	0.2	i
ARG	160	48.75	43.71	6.51	42.23	21.6	
LYS	161	70.91	60.22	9.18	61.73	37.5	
LEU	162	81.01	37.40	21.97	59.04	40.4	

(a) (b)

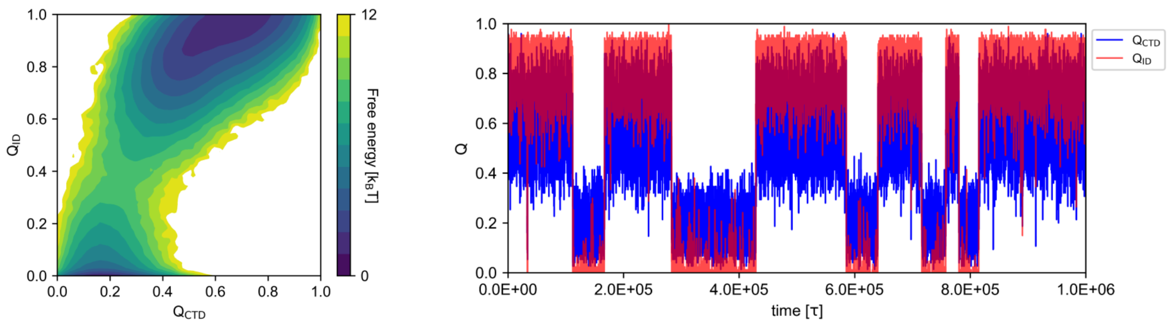

Figure S1. Concurrent NTD-CTD dissociation and CTD unfolding, as ascertained by all-atom SBM simulations of RfaH in the autoinhibited state. (a) Two-dimensional folding landscape of RfaH CTD at T = $0.92T_F$, using Q_{CTD} and Q_{ID} as reaction coordinates. A two-state, concurrent unfolding of the CTD upon dissociation from the NTD is observed. (b) Representative trajectory at T = $0.92T_F$, illustrating the changes in Q_{CTD} and Q_{ID} during the simulation.

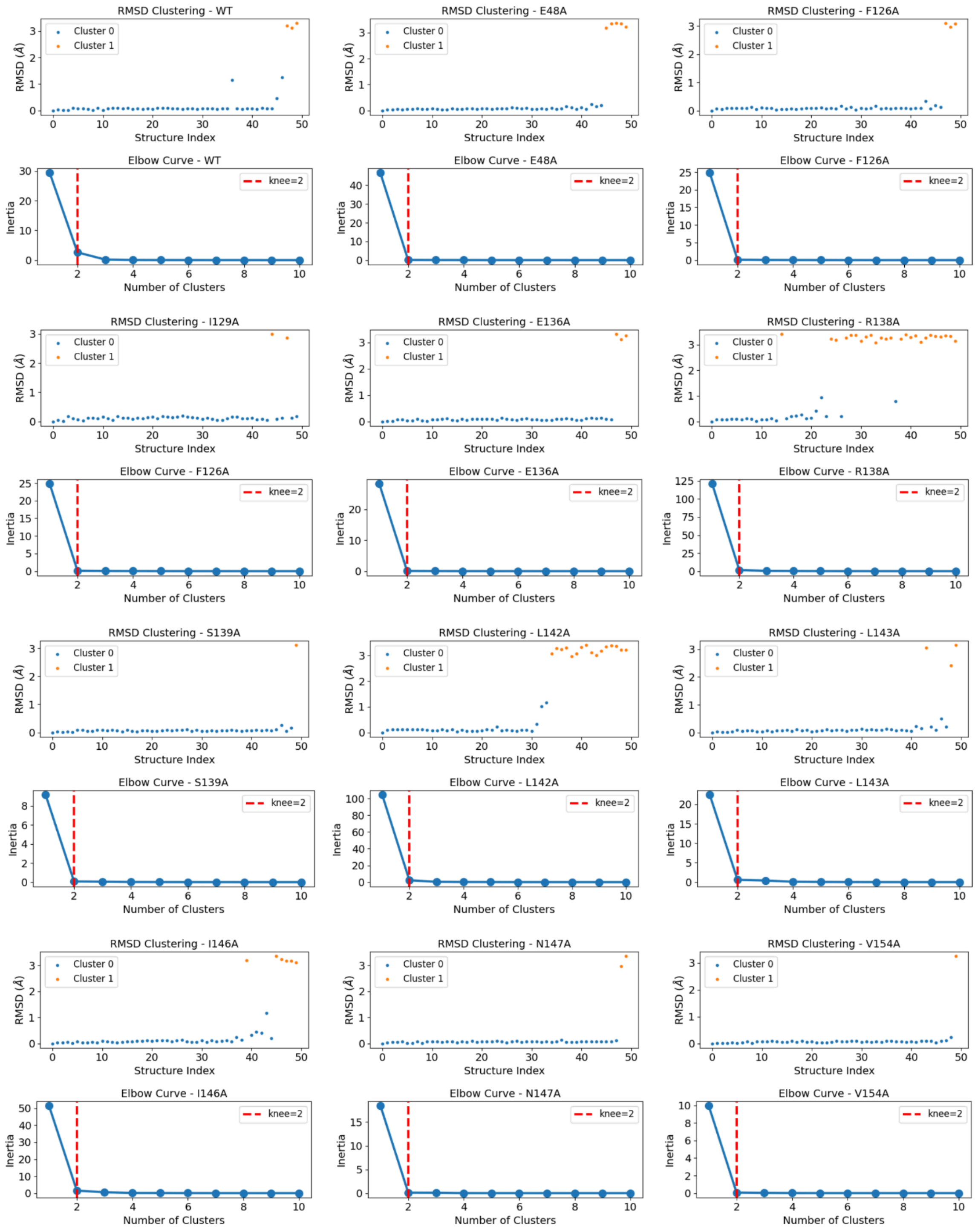

Figure S2. *K*-means clustering of all models for all RfaH variants generated using ColabFold (5 model parameters, 10 seeds, 3 recycles, no dropouts) based on the RMSD of CTD residues 126-131. Each RMSD clustering plot against structure rank 1 is accompanied by the elbow curve to determine the optimal number of clusters per variant.

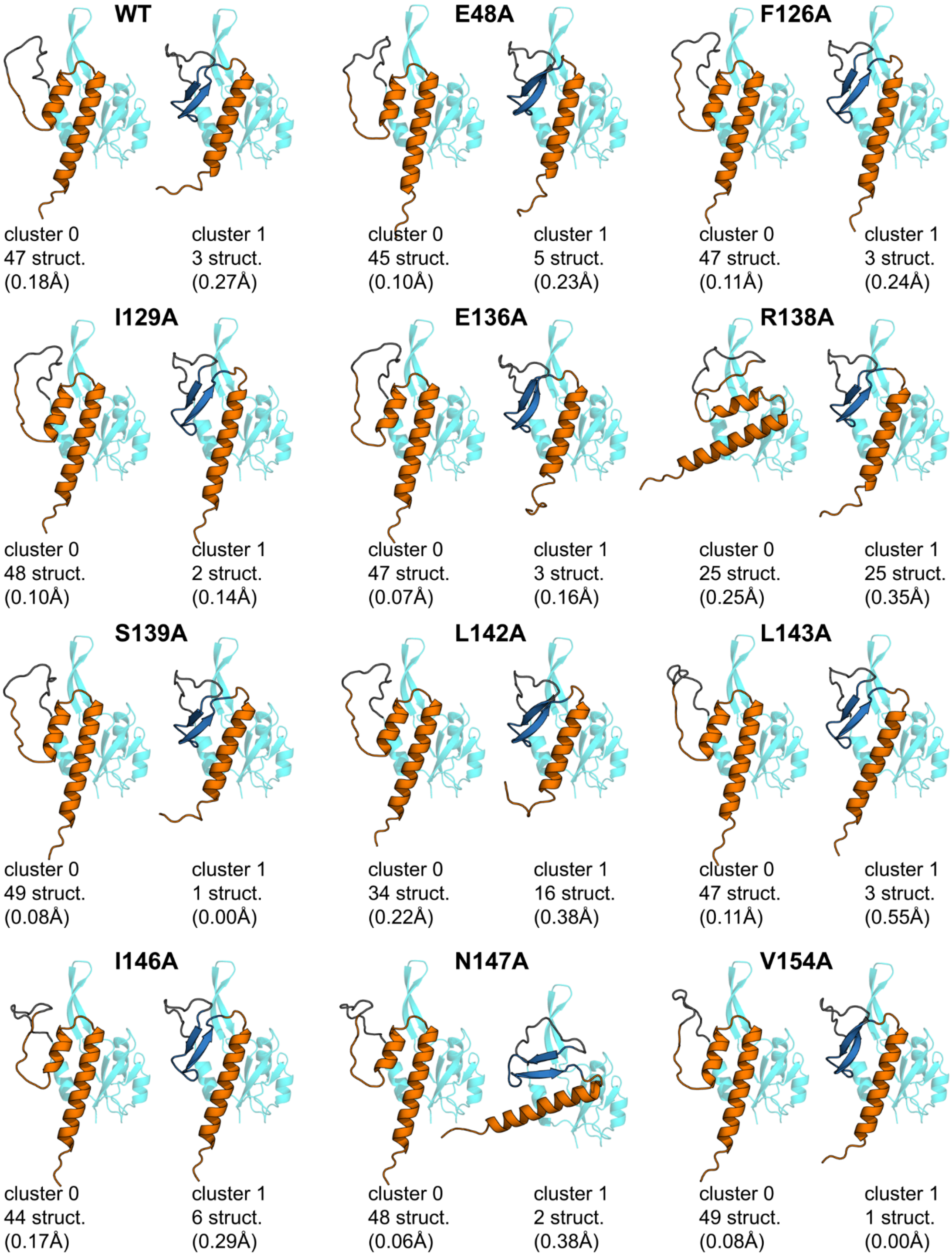

Figure S3. Representative structures of each cluster (5 model parameters, 10 seeds, 3 recycles, no dropouts) for each RfaH variant were determined using *k*-means clustering. The NTD is represented in cyan, the CTD in orange, and the β-strand CTD region (residues 114-131) in blue when present. The number of structures per cluster is indicated below. The number in parentheses is the average RMSD of residues 126-131 in each cluster.

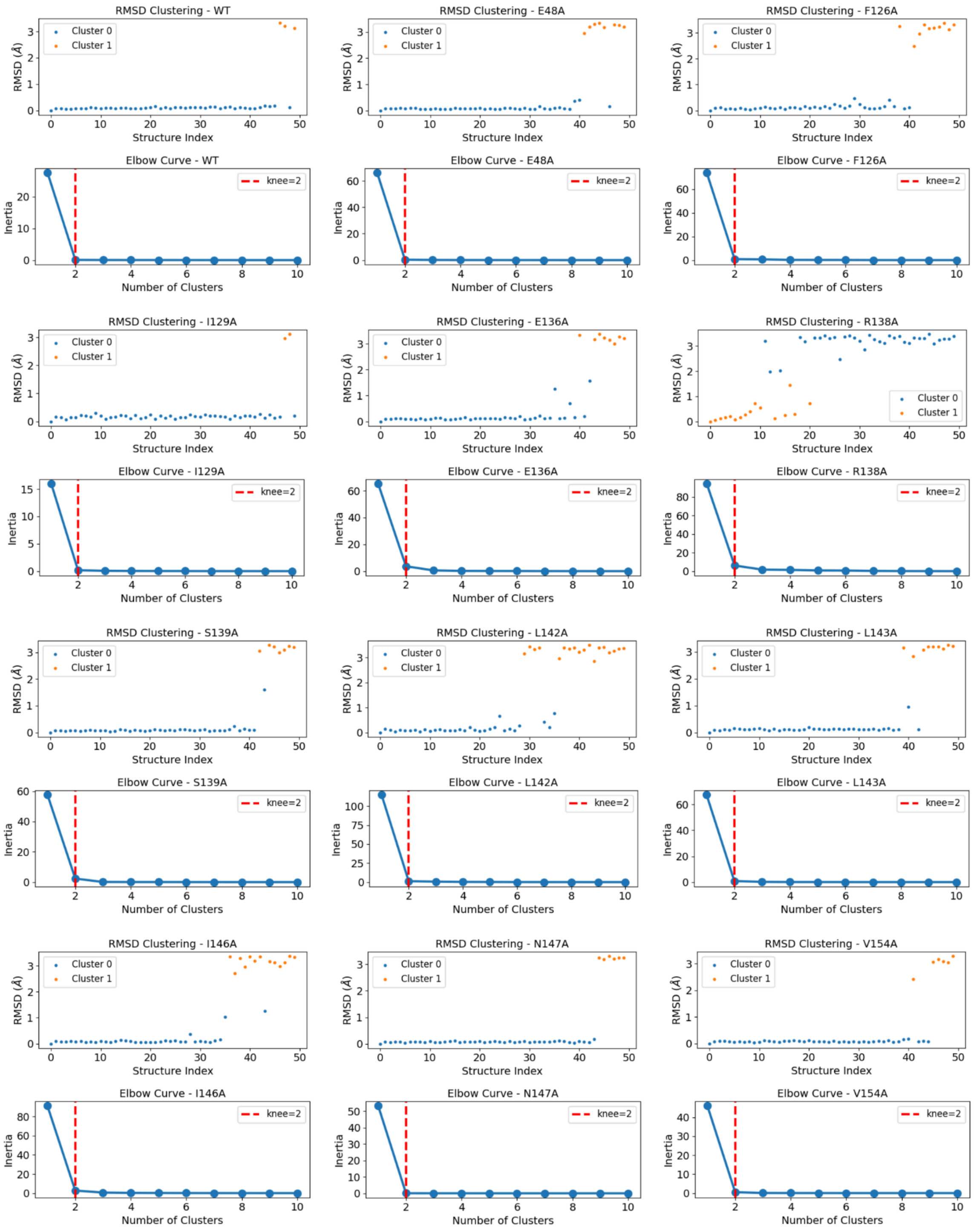

Figure S4. *K*-means clustering of all models for all RfaH variants generated using ColabFold (5 model parameters, 10 seeds, 3 recycles, with dropouts) based on the RMSD of CTD residues 126-131. Each RMSD clustering plot against structure rank 1 is accompanied by the elbow curve to determine the optimal number of clusters per variant.

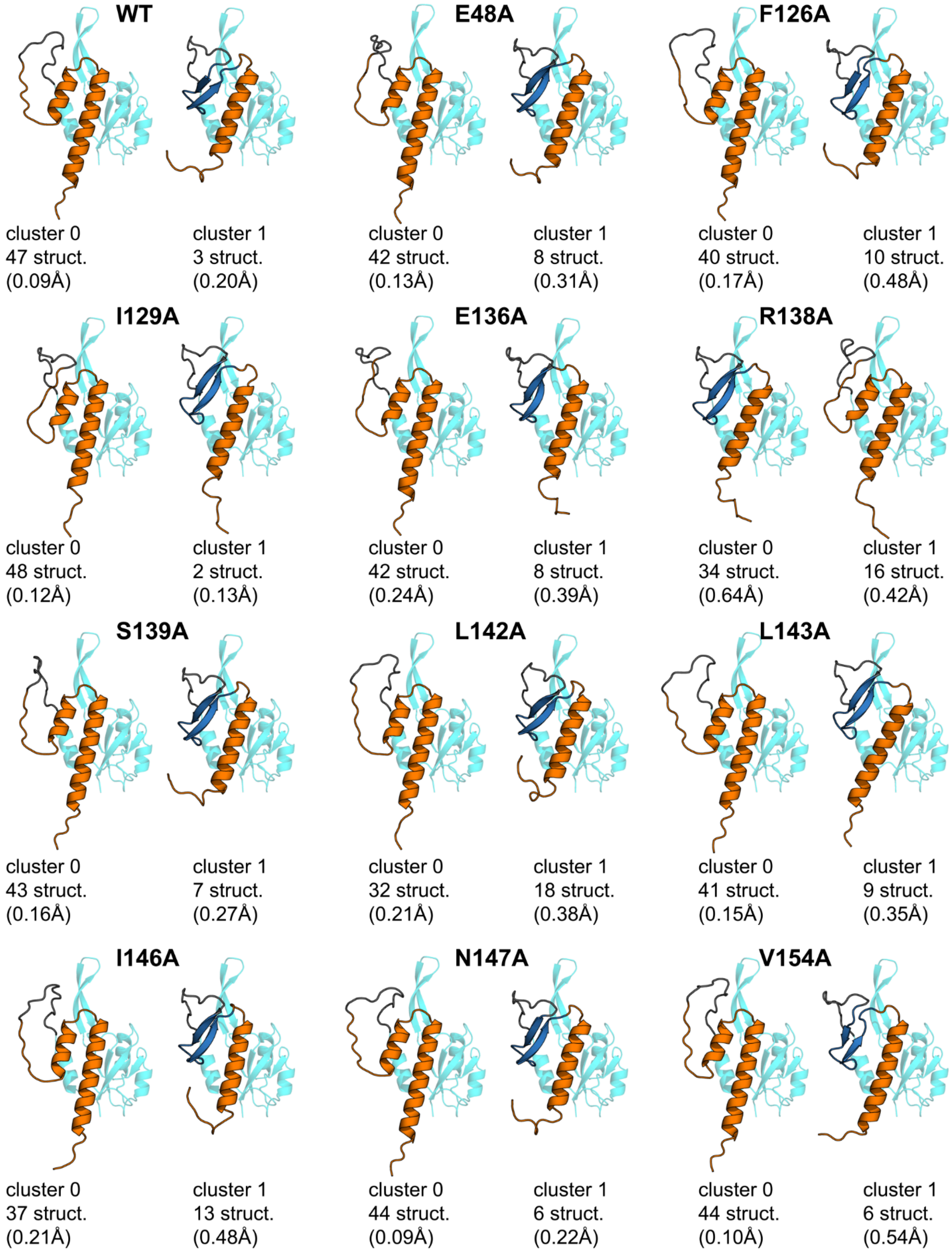

Figure S5. Representative structures of each cluster (5 model parameters, 10 seeds, 3 recycles, with dropouts) for each RfaH variant using *k*-means clustering. The NTD is represented in cyan, the CTD in orange, and the β-strand CTD region (residues 114-131) in blue when present. The number of structures per cluster is indicated below. The number in parentheses is the average RMSD of residues 126-131 in each cluster.

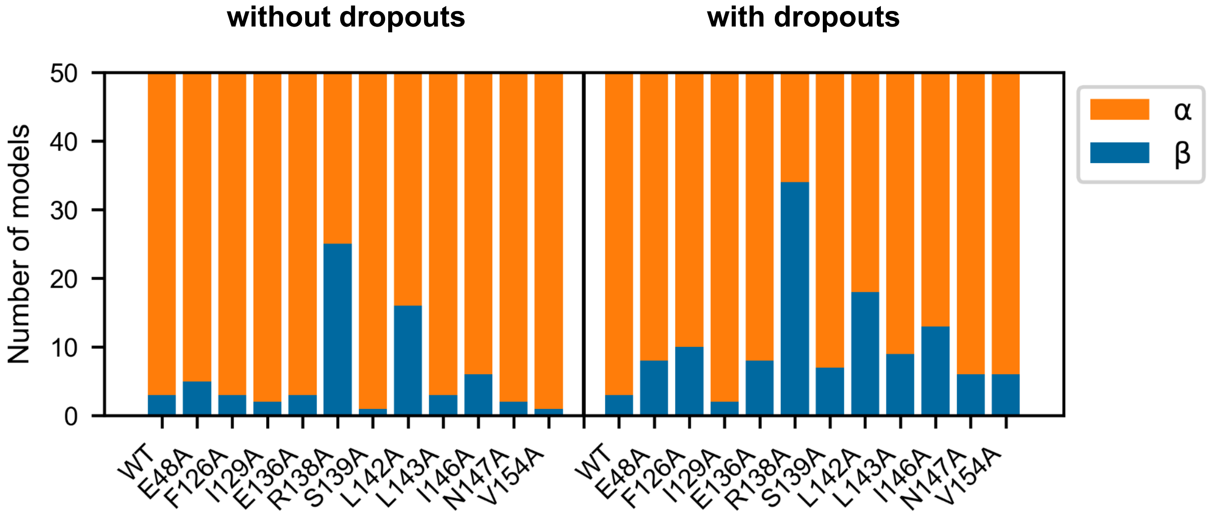

Figure S6. Counting of the number of predicted structures with either α-helical (orange) or β-strand (blue) content in the region 126-131 of the CTD of all RfaH variants generated using ColabFold, based on the results from *k*-means clustering. For all protein structure predictions, ColabFold was run without using structural templates, using 10 random seeds, 5 model parameters, and 3 recycles, thus generating 50 models per input sequence, either with or without dropouts.

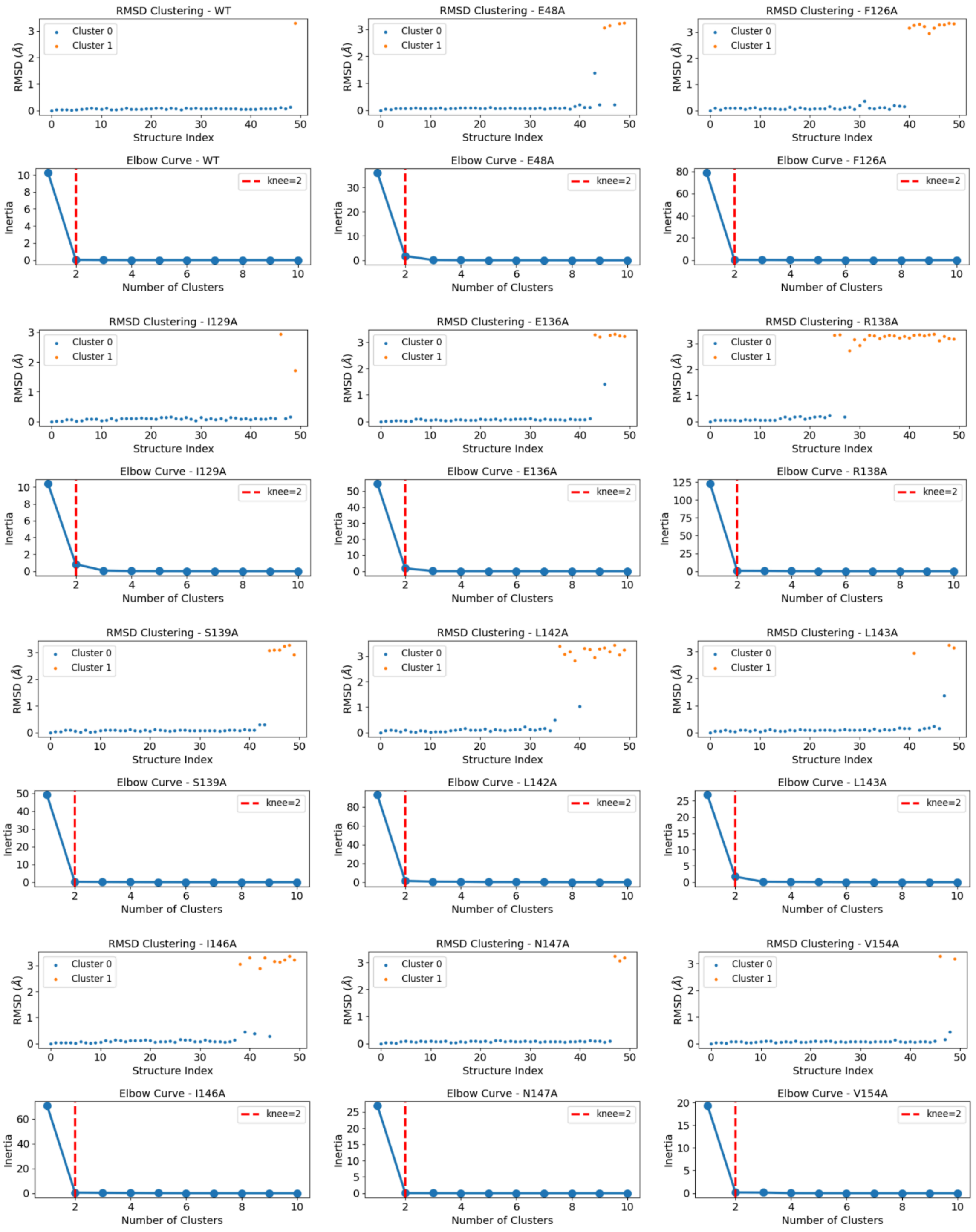

Figure S7. *K*-means clustering of all models for all RfaH variants generated using ColabFold (5 model parameters, 10 seeds, 12 recycles, no dropouts) based on the RMSD of CTD residues 126-131. Each RMSD clustering plot against structure rank 1 is accompanied by the elbow curve to determine the optimal number of clusters per variant.

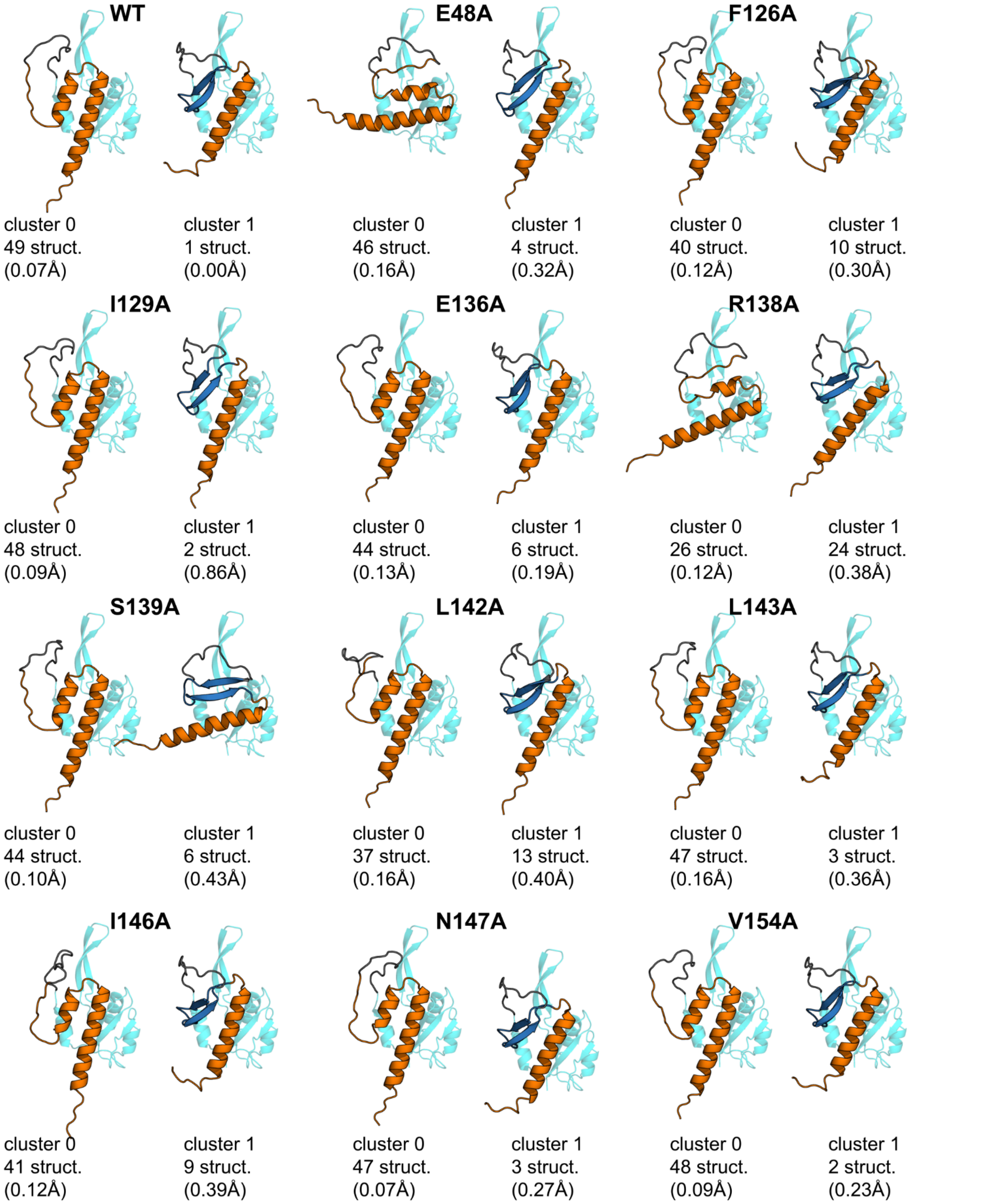

Figure S8. Representative structures of each cluster (5 model parameters, 10 seeds, 12 recycles, no dropouts) for each RfaH variant were determined using *k*-means clustering. The NTD is represented in cyan, the CTD in orange, and the β-strand CTD region (residues 114-131) in blue when present. The number of structures per cluster is indicated below. The number in parentheses is the average RMSD of residues 126-131 in each cluster.

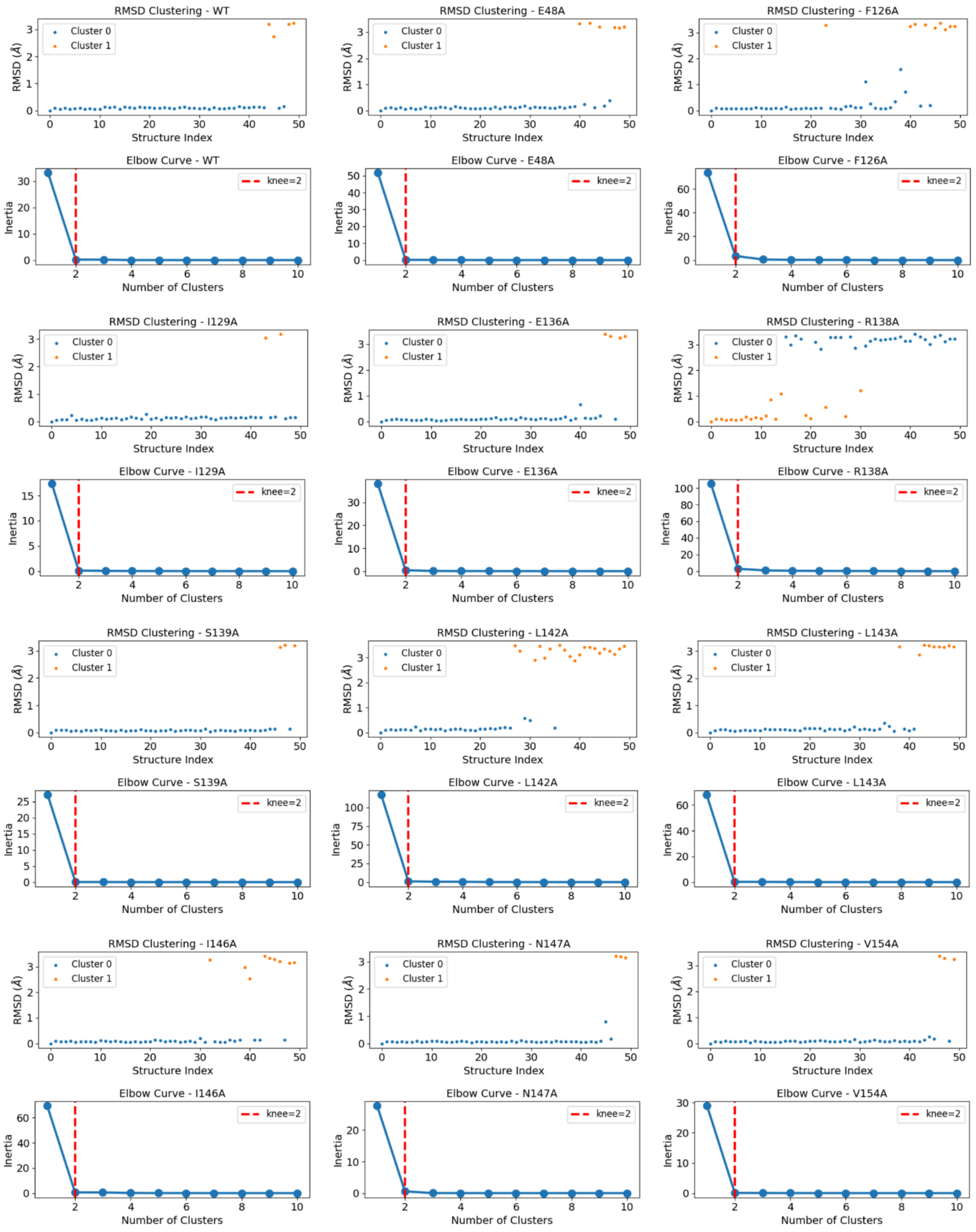

Figure S9. *K*-means clustering of all models for all RfaH variants generated using ColabFold (5 model parameters, 10 seeds, 12 recycles, with dropouts) based on the RMSD of CTD residues 126-131. Each RMSD clustering plot against structure rank 1 is accompanied by the elbow curve to determine the optimal number of clusters per variant.

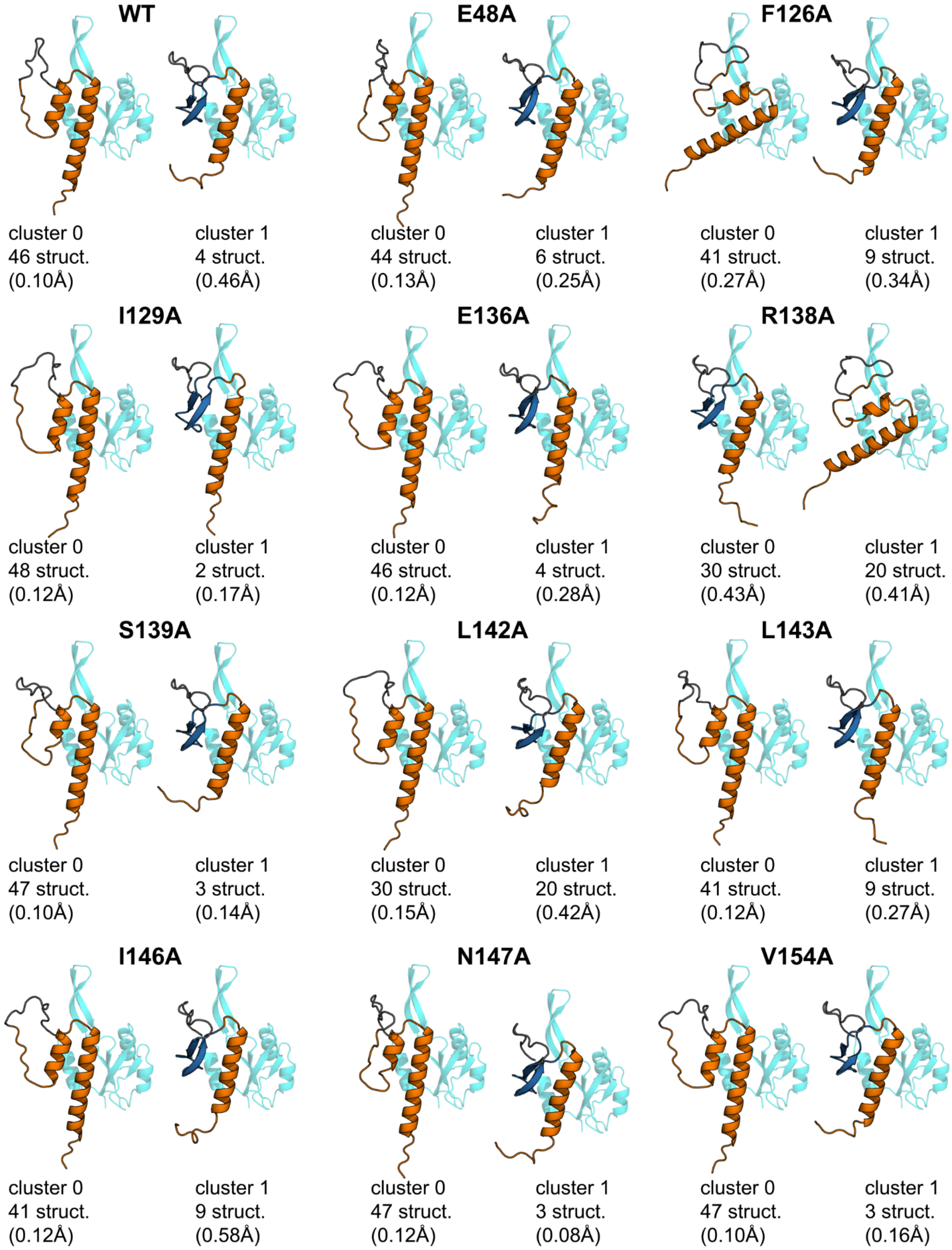

Figure S10. Representative structures of each cluster (5 model parameters, 10 seeds, 12 recycles, with dropouts) for each RfaH variant using *k*-means clustering. The NTD is represented in cyan, the CTD in orange, and the β-strand CTD region (residues 114-131) in blue when present. The number of structures per cluster is indicated below. The number in parentheses is the average RMSD of residues 126-131 in each cluster.

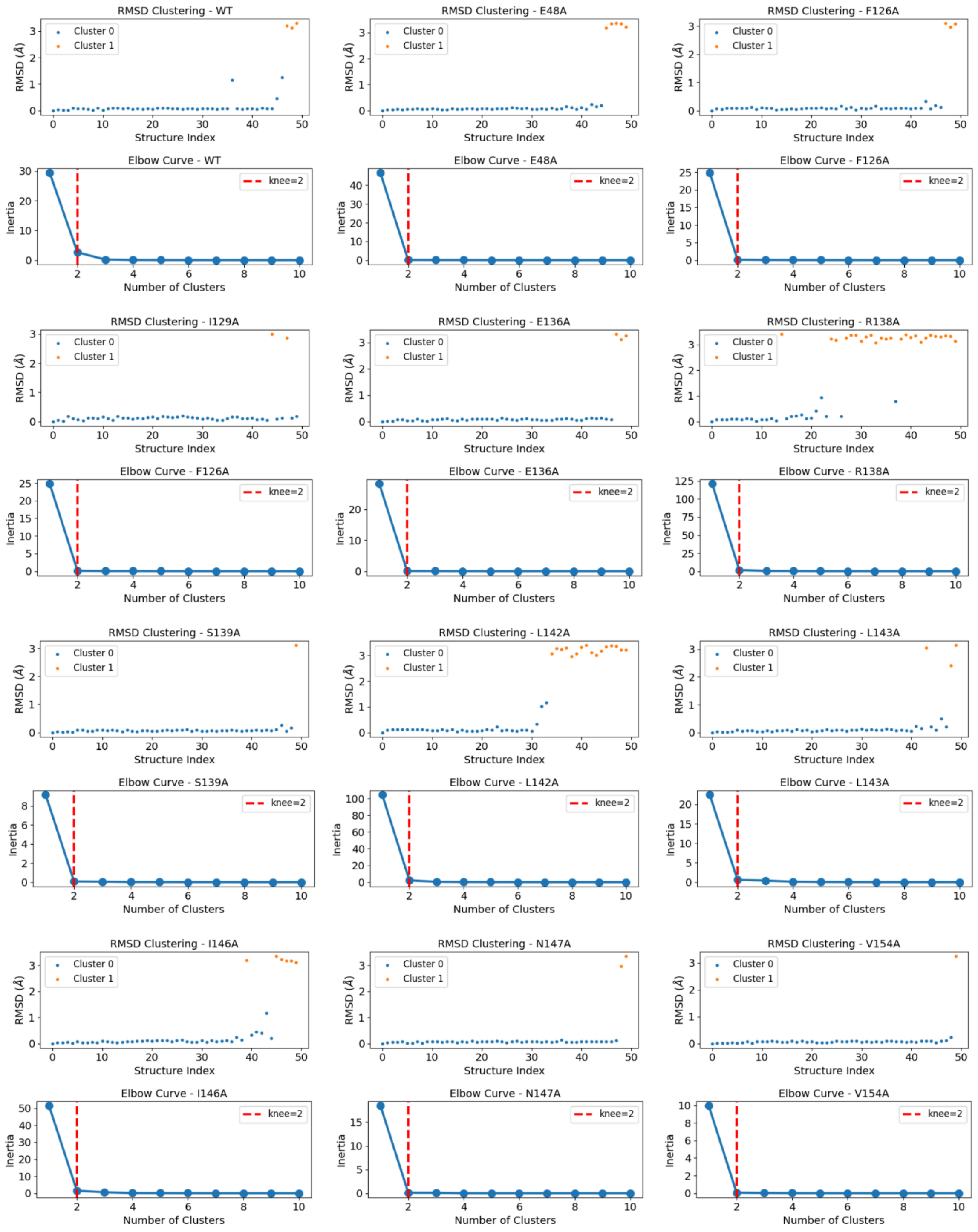

Figure S11. *K*-means clustering of all models for all RfaH variants generated using ColabFold (5 model parameters, 10 seeds, 3 recycles, no dropouts, same MSA as RfaH WT) based on the RMSD of CTD residues 126-131. Each RMSD clustering plot against structure rank 1 is accompanied by the elbow curve to determine the optimal number of clusters per variant.

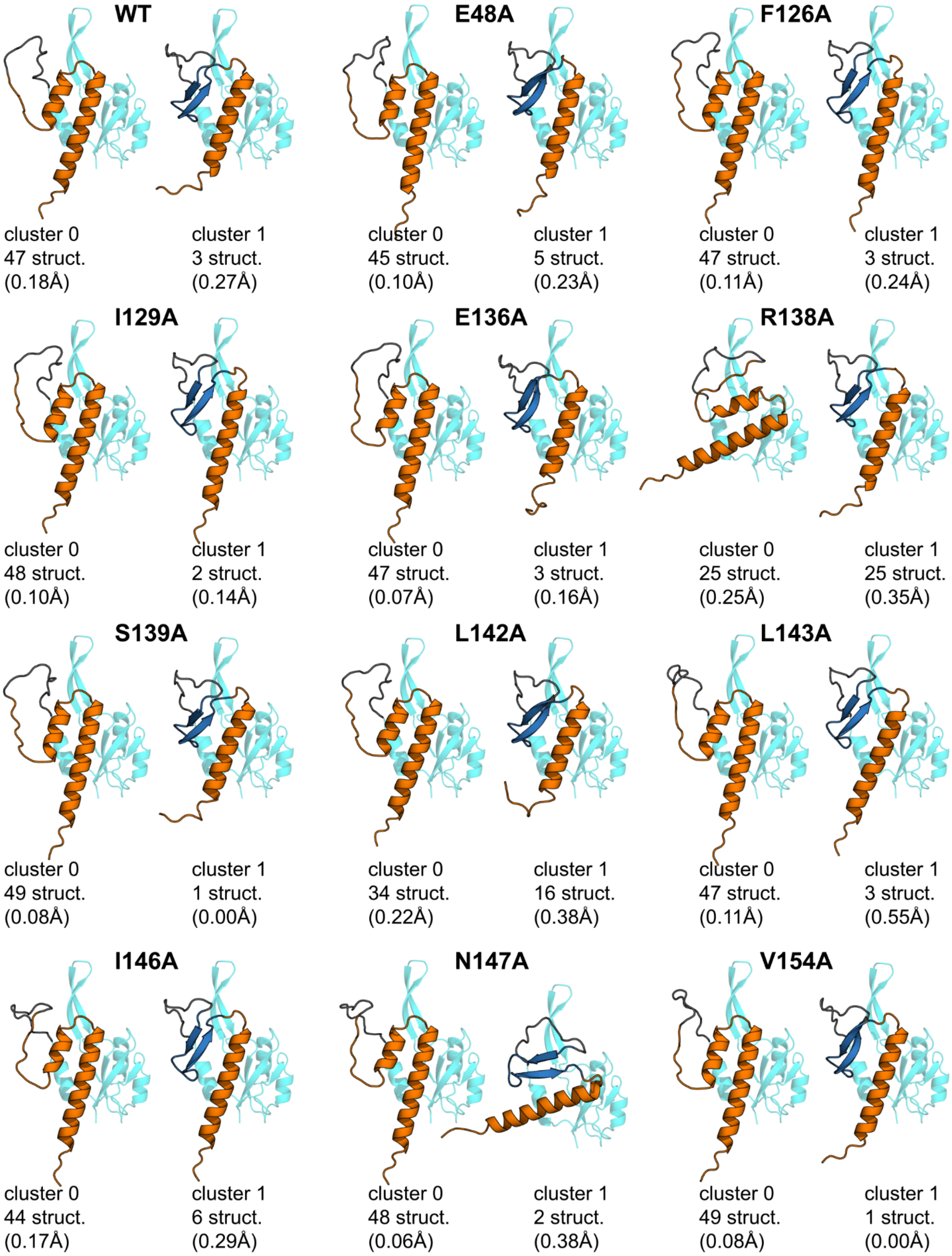

Figure S12. Representative structures of each cluster (5 model parameters, 10 seeds, 3 recycles, no dropouts, same MSA as RfaH WT) for each RfaH variant using *k*-means clustering. The NTD is represented in cyan, the CTD in orange, and the β-strand CTD region (residues 114-131) in blue when present. The number of structures per cluster is indicated below. The number in parentheses is the average RMSD of residues 126-131 in each cluster.

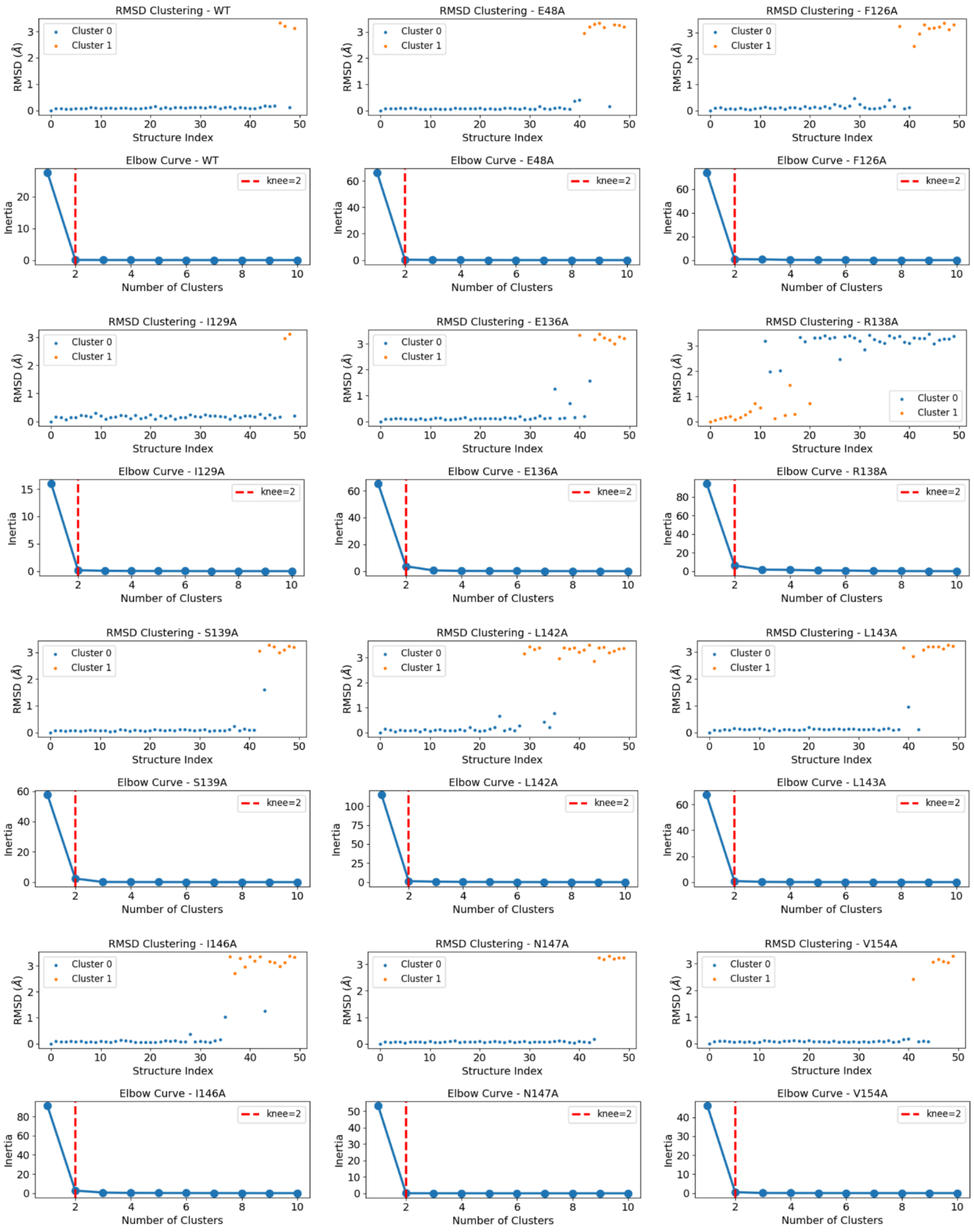

Figure S13. *K*-means clustering of all models for all RfaH variants generated using ColabFold (5 model parameters, 10 seeds, 3 recycles, with dropouts, same MSA as RfaH WT) based on the RMSD of CTD residues 126-131. Each RMSD clustering plot against structure rank 1 is accompanied by the elbow curve to determine the optimal number of clusters per variant.

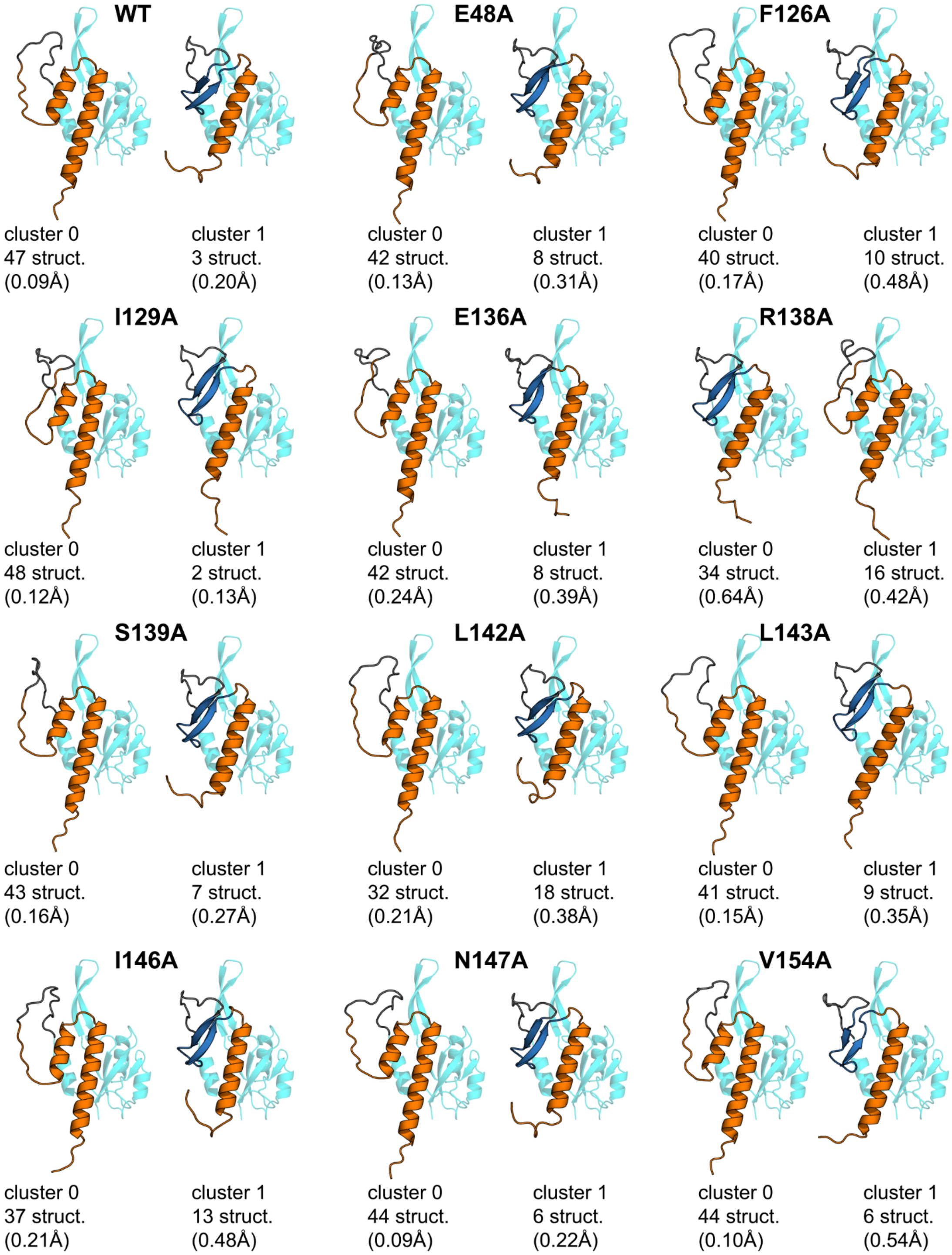

Figure S14. Representative structures of each cluster (5 model parameters, 10 seeds, 3 recycles, with dropouts, same MSA as RfaH WT) for each RfaH variant using *k*-means clustering. The NTD is represented in cyan, the CTD in orange, and the β-strand CTD region (residues 114-131) in blue when present. The number of structures per cluster is indicated below. The number in parentheses is the average RMSD of residues 126-131 in each cluster.

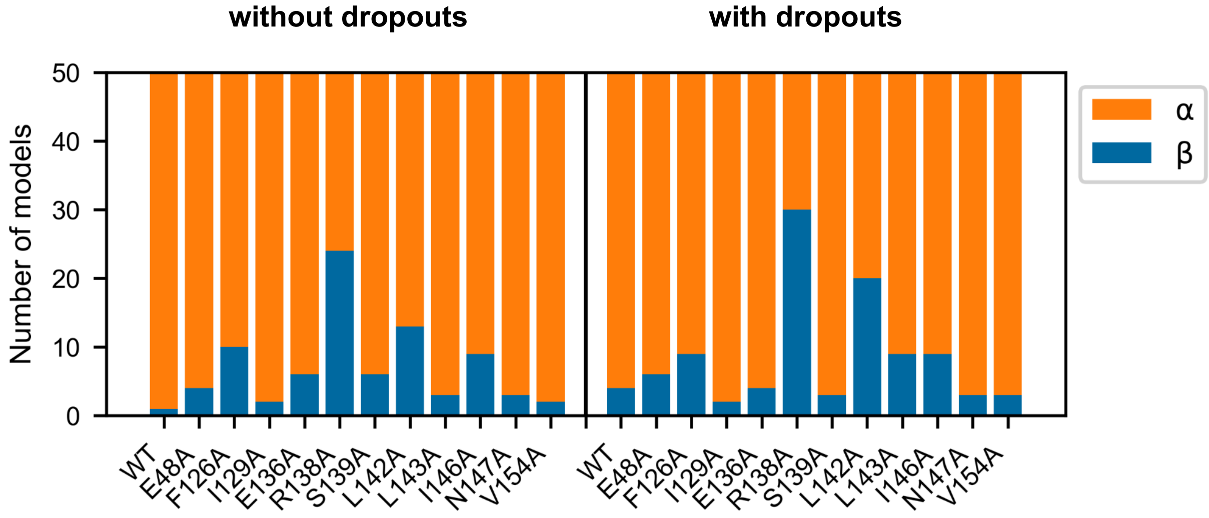

Figure S15. Counting the number of predicted structures with either α-helical (orange) or β-strand (blue) content in the region 126-131 of the CTD of all RfaH variants generated using ColabFold, based on the results from *k*-means clustering. For all protein structure predictions, ColabFold was run without using structural templates, using 10 random seeds, 5 model parameters, 3 recycles, and the same MSA as RfaH WT, thus generating 50 models per input sequence, either without or with dropouts.

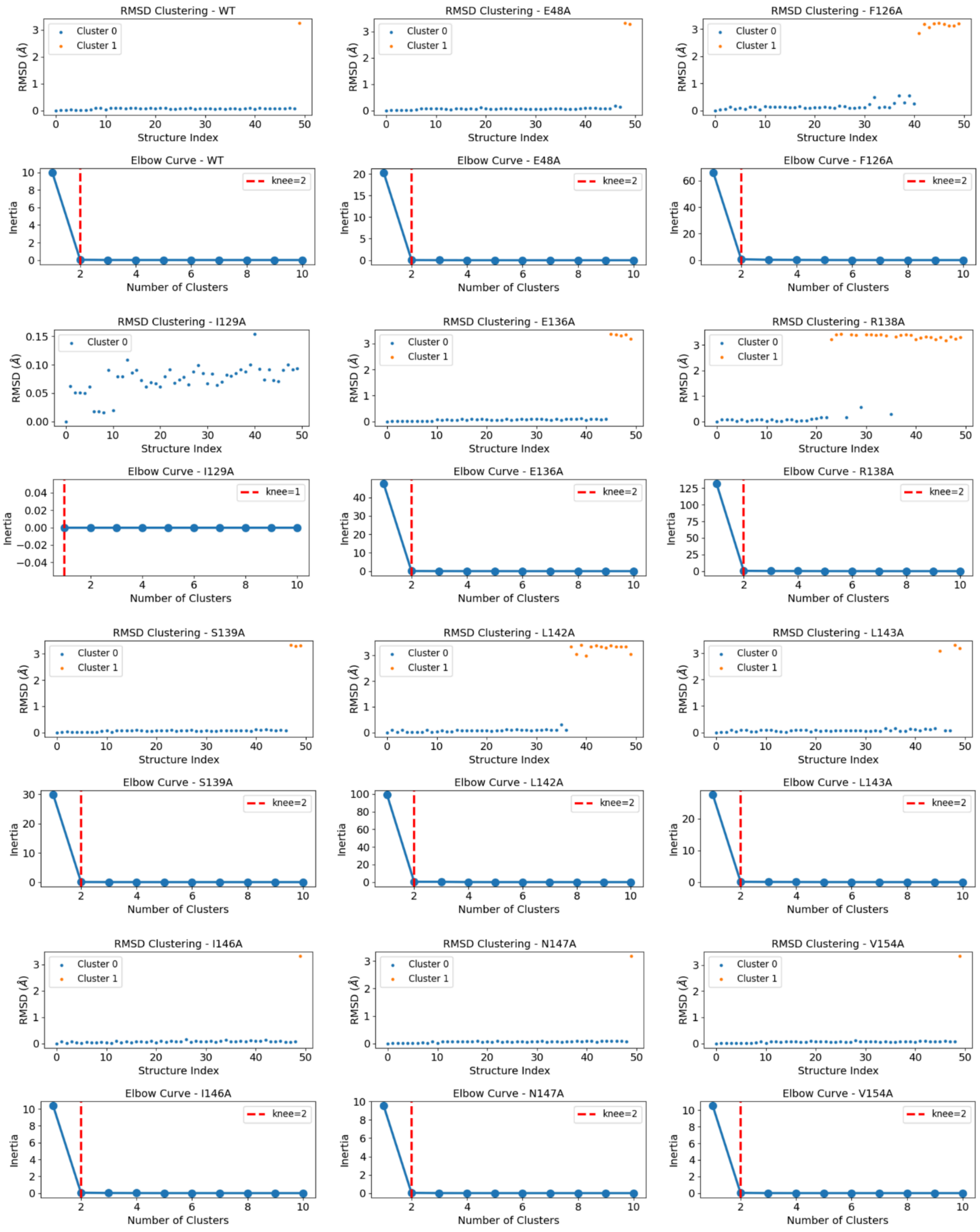

Figure S16. *K*-means clustering of all models for all RfaH variants generated using ColabFold (5 model parameters, 10 seeds, 12 recycles, no dropouts, same MSA as RfaH WT) based on the RMSD of CTD residues 126-131. Each RMSD clustering plot against structure rank 1 is accompanied by the elbow curve to determine the optimal number of clusters per variant.

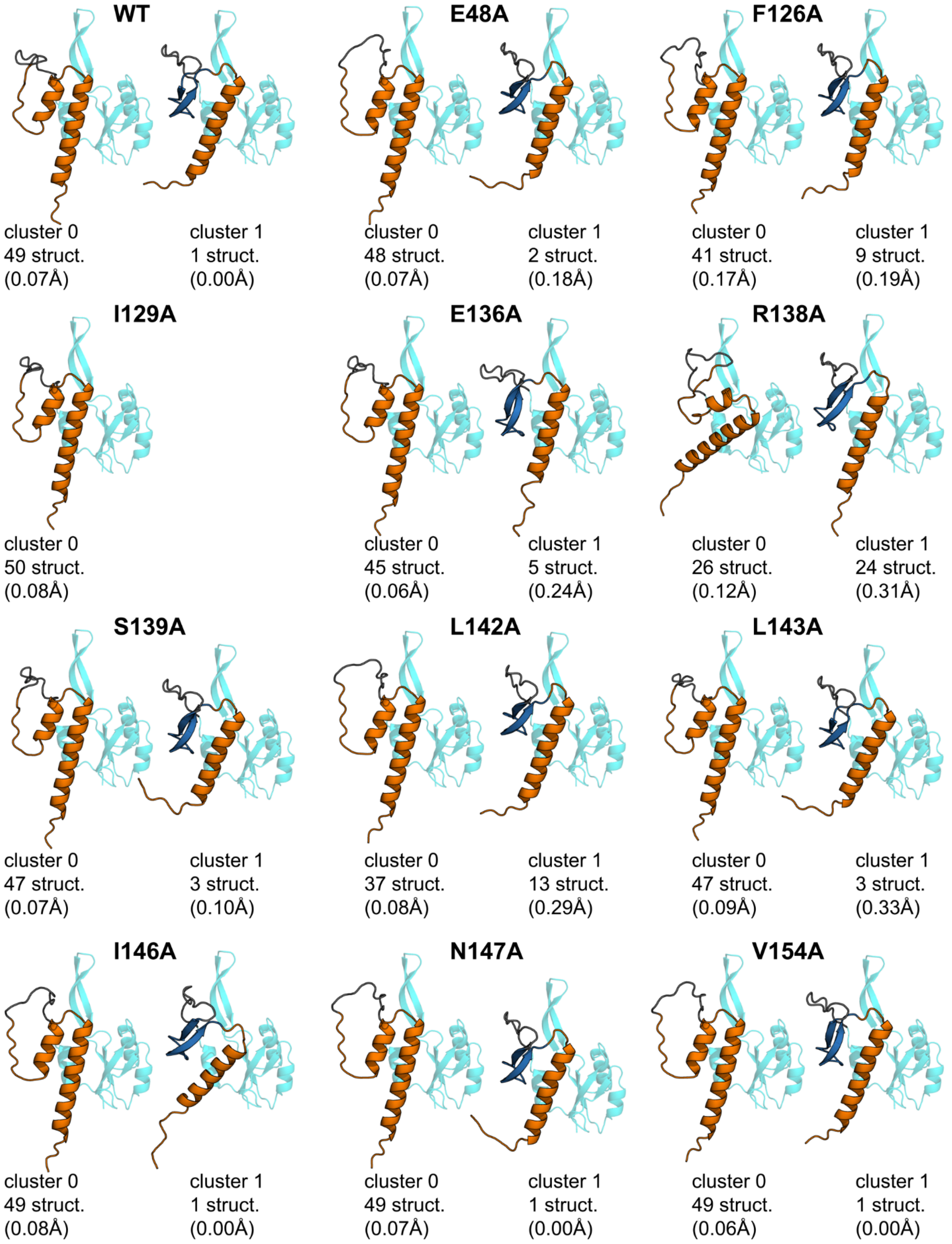

Figure S17. Representative structures of each cluster (5 model parameters, 10 seeds, 12 recycles, no dropouts, same MSA as RfaH WT) for each RfaH variant using *k*-means clustering. The NTD is represented in cyan, the CTD in orange, and the β-strand CTD region (residues 114-131) in blue when present. The number of structures per cluster is indicated below. The number in parentheses is the average RMSD of residues 126-131 in each cluster.

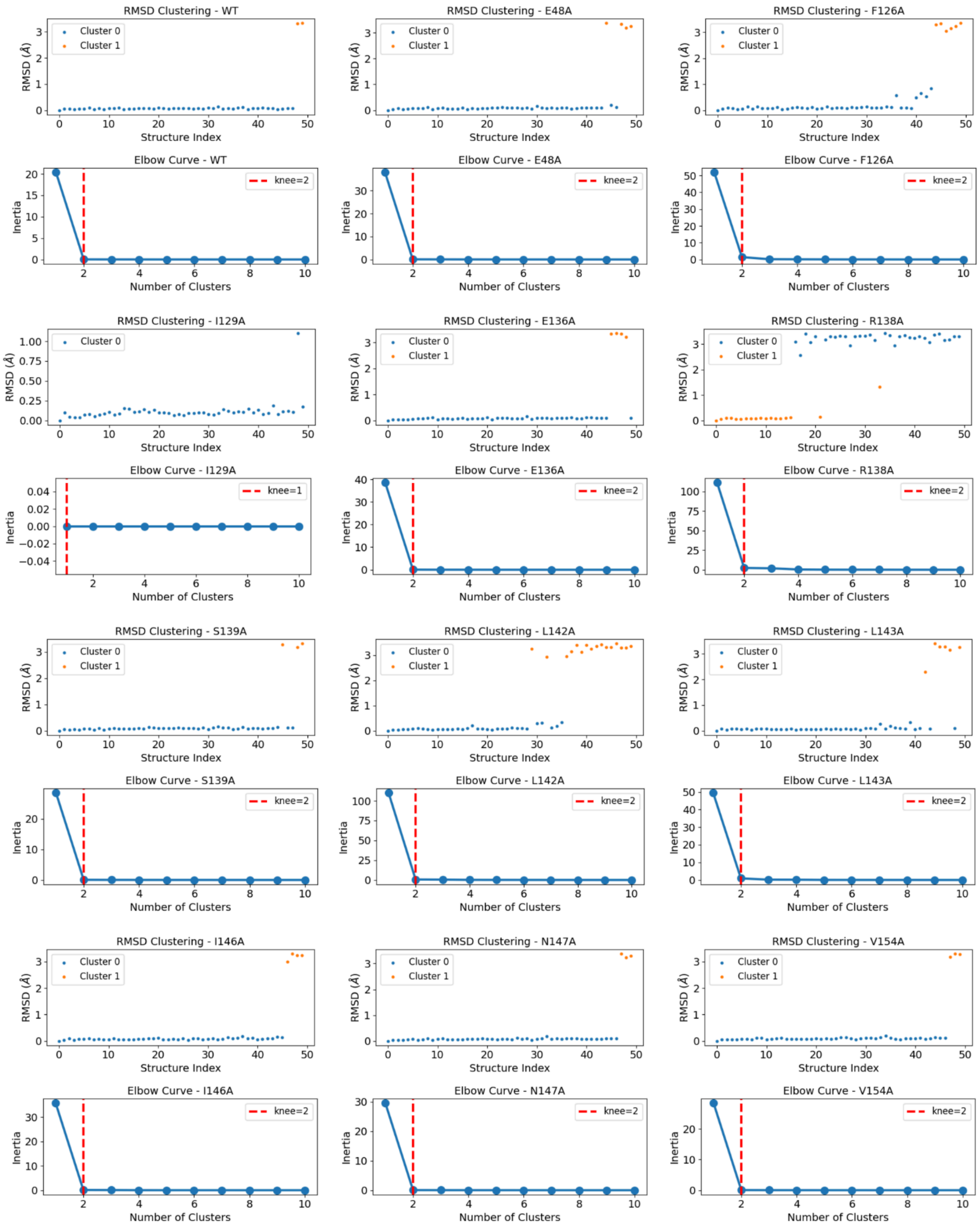

Figure S18. *K*-means clustering of all models for all RfaH variants generated using ColabFold (5 model parameters, 10 seeds, 12 recycles, with dropouts, same MSA as RfaH WT) based on the RMSD of CTD residues 126-131. Each RMSD clustering plot against structure rank 1 is accompanied by the elbow curve to determine the optimal number of clusters per variant.

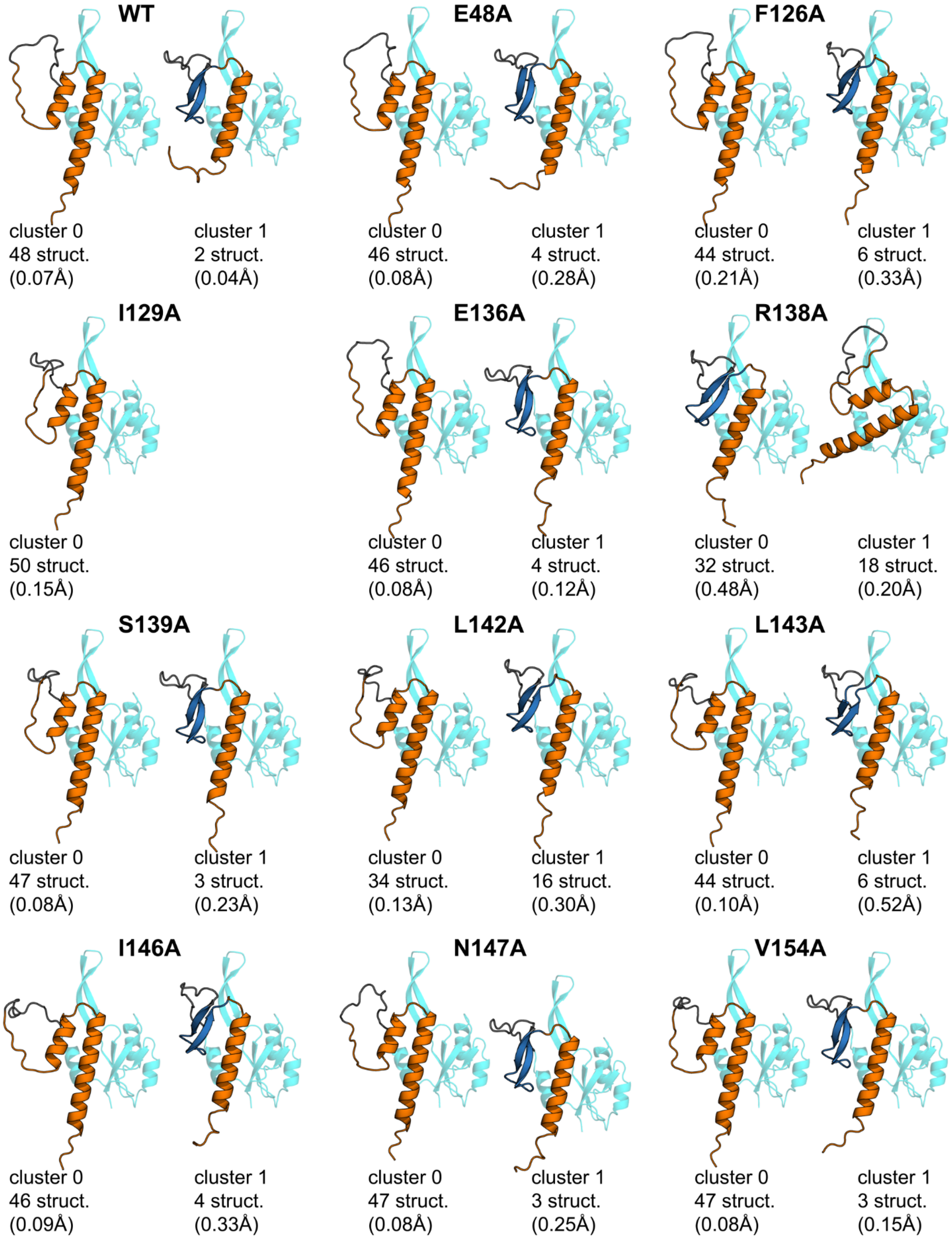

Figure S19. Representative structures of each cluster (5 model parameters, 10 seeds, 12 recycles, with dropouts, same MSA as RfaH WT) for each RfaH variant using *k*-means clustering. The NTD is represented in cyan, the CTD in orange, and the β-strand CTD region (residues 114-131) in blue when present. The number of structures per cluster is indicated below. The number in parentheses is the average RMSD of residues 126-131 in each cluster.

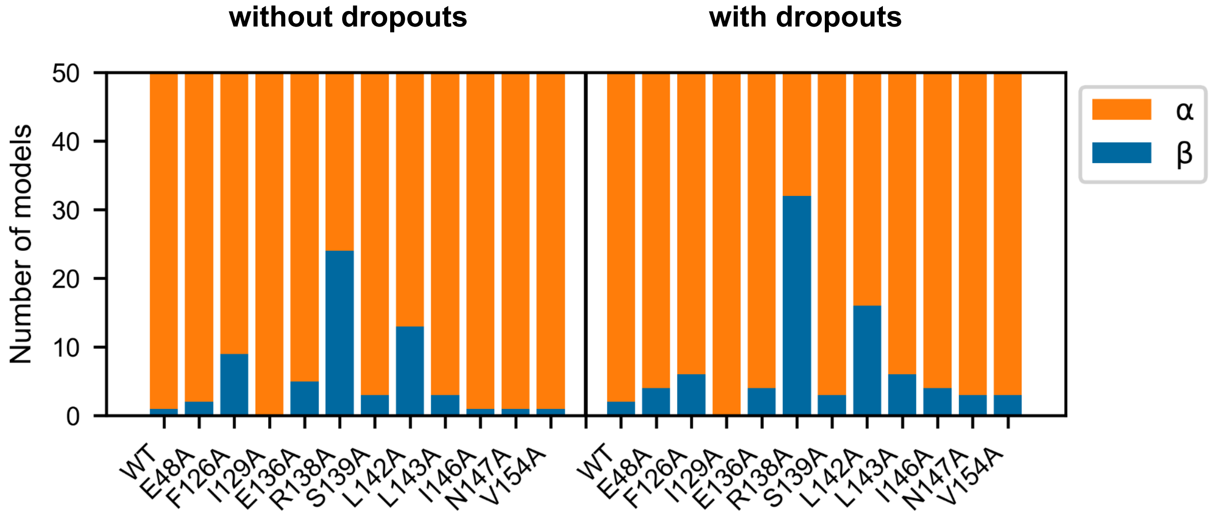

Figure S20. Counting the number of predicted structures with either α-helical (orange) or β-strand (blue) content in the region 126-131 of the CTD of all RfaH variants generated using ColabFold, based on the results from *k*-means clustering. For all protein structure predictions, ColabFold was run without using structural templates, using 10 random seeds, 5 model parameters, 12 recycles, and the same MSA as RfaH WT, thus generating 50 models per input sequence, either without or with dropouts.

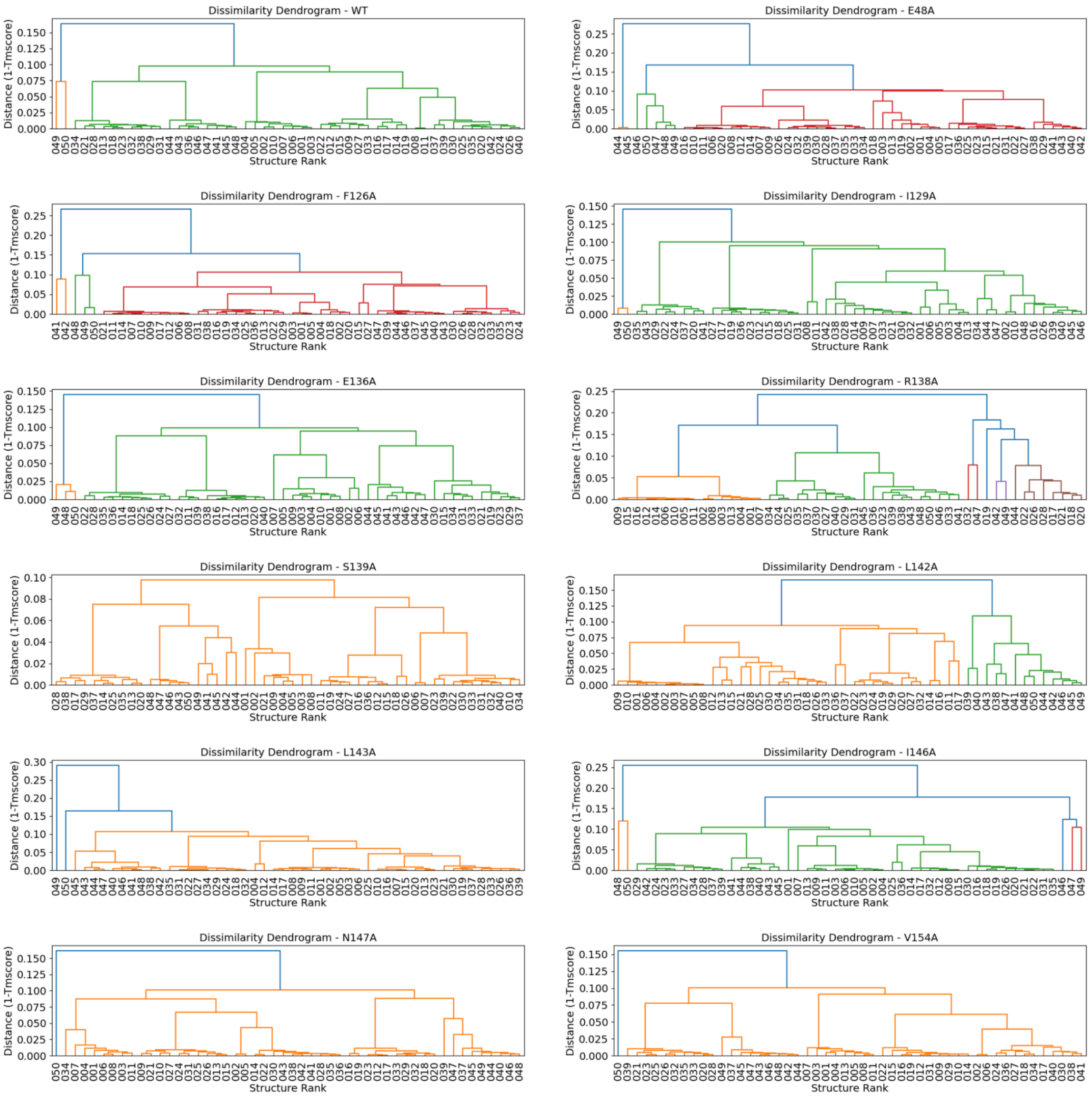

Figure S21. Hierarchical clustering based on the dissimilarity (1 - TM-score) of all predicted structures generated for all variants using ColabFold (5 model parameters, 10 seeds, 12 recycles, no dropouts) against the structure ranked as 1 for each variant. A distance of 0.12 was considered as optimal to cluster the structures in different groups based on their TM-score against the best predicted structure. The different colors in the dendrograms represent the different clusters for each variant.

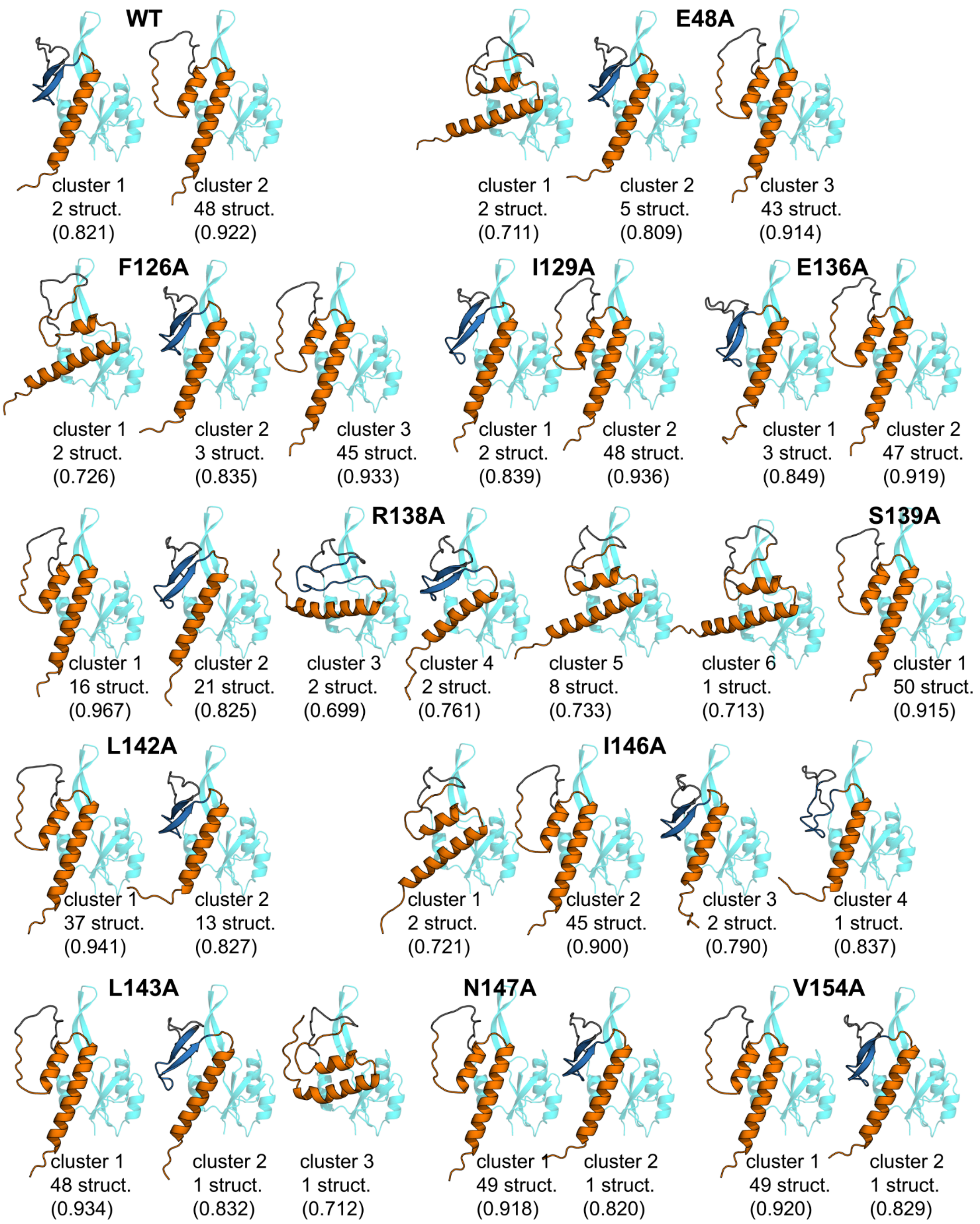

Figure S22. Representative structures of each cluster (5 model parameters, 10 seeds, 12 recycles, no dropouts) for each RfaH variant using hierarchical clustering based on TM-score. The NTD is represented in cyan, the CTD in orange, and the β-strand CTD region (residues 114-131) in blue when present. The number of structures per cluster is indicated below. The number in parentheses is the average TM-score of each cluster against the best predicted structure (rank 1) for each variant.

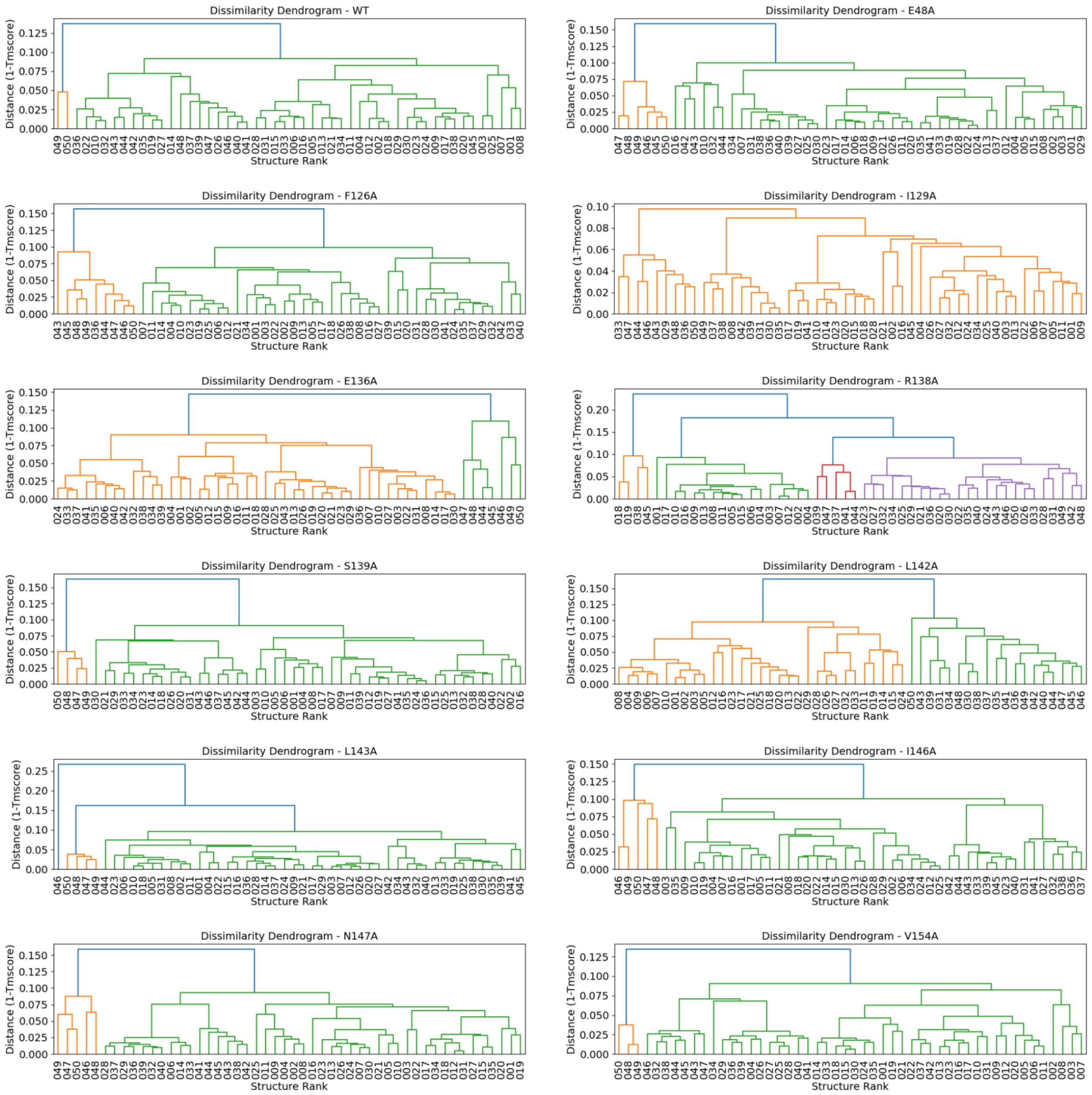

Figure S23. Hierarchical clustering based on the dissimilarity (1 - TM-score) of all predicted structures generated for all variants using ColabFold (5 model parameters, 10 seeds, 12 recycles, with dropouts) against the structure ranked as 1 for each variant. A distance of 0.12 was considered as optimal to cluster the structures in different groups based on their TM-score against the best predicted structure. The different colors in the dendrograms represent the different clusters for each variant.

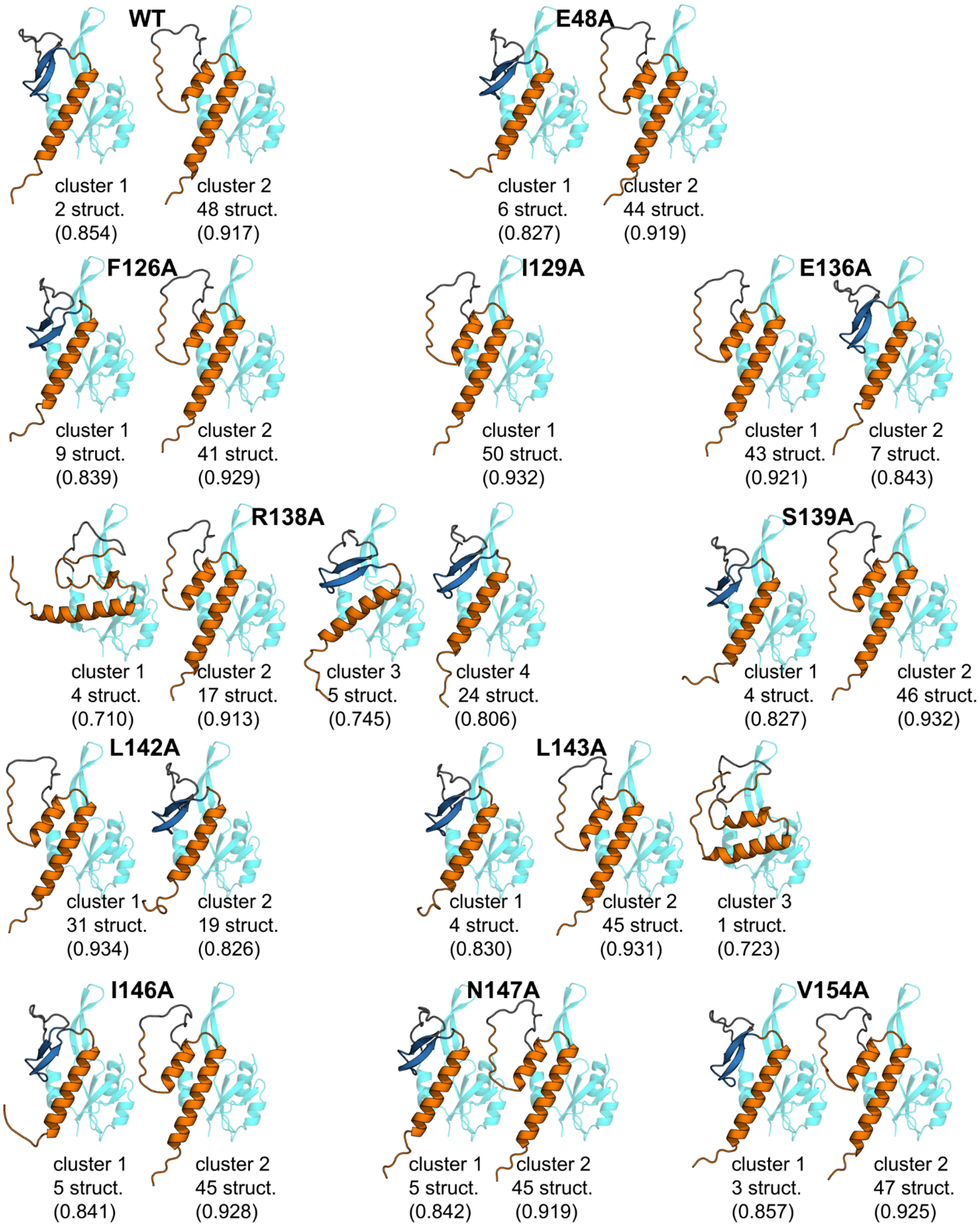

Figure S24. Representative structures of each cluster (5 model parameters, 10 seeds, 12 recycles, with dropouts) for each RfaH variant using hierarchical clustering based on TM-score. The NTD is represented in cyan, the CTD in orange, and the β-strand CTD region (residues 114-131) in blue when present. The number of structures per cluster is indicated below. The number in parentheses is the average TM-score of each cluster against the best predicted structure (rank 1) for each variant.

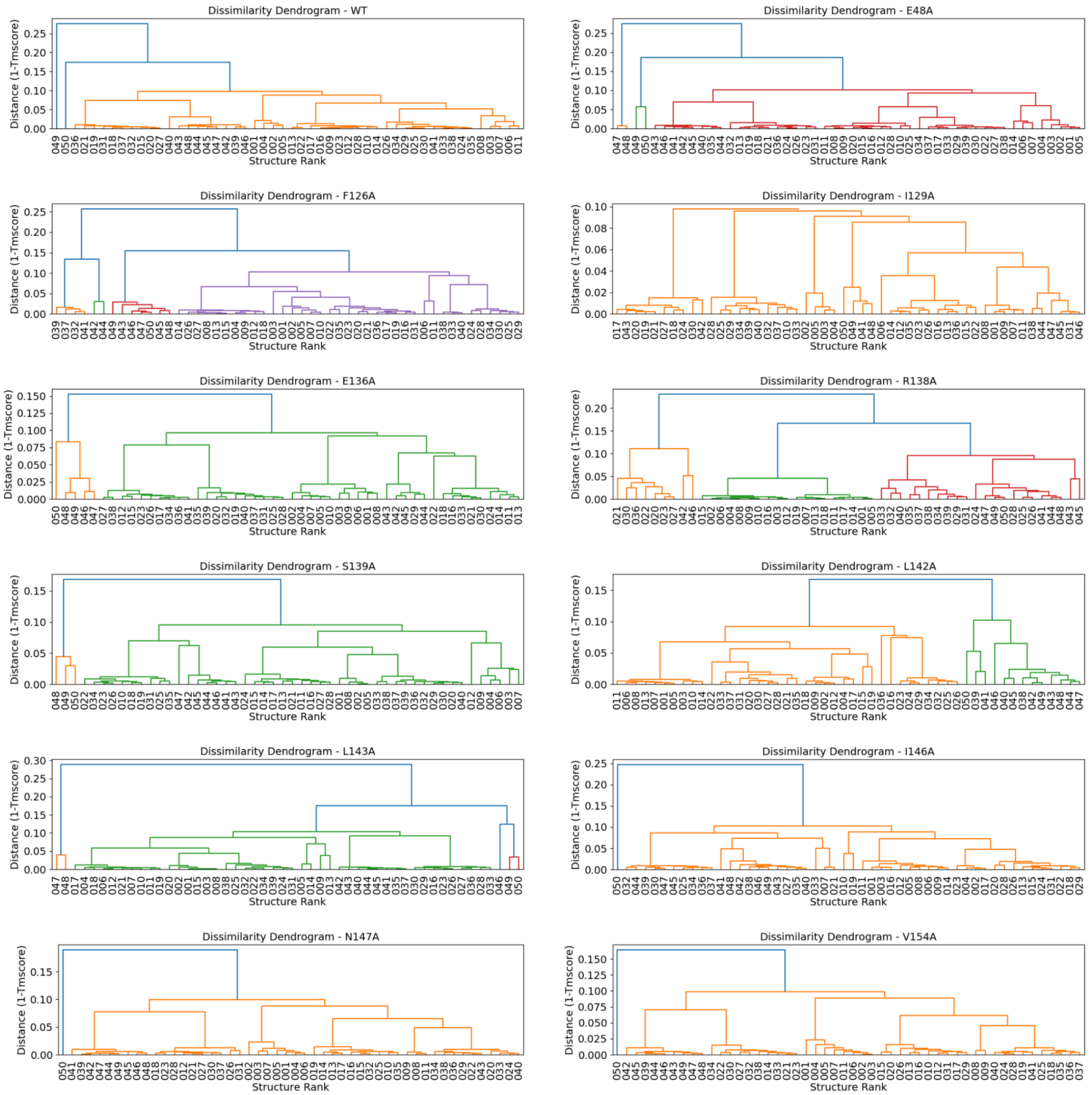

Figure S25. Hierarchical clustering based on the dissimilarity (1 - TM-score) of all predicted structures generated for all variants using ColabFold (5 model parameters, 10 seeds, 12 recycles, no dropouts, same MSA as RfaH WT) against the structure ranked as 1 for each variant. A distance of 0.12 was considered as optimal to cluster the structures in different groups based on their TM-score against the best predicted structure. The different colors in the dendrograms represent the different clusters for each variant.

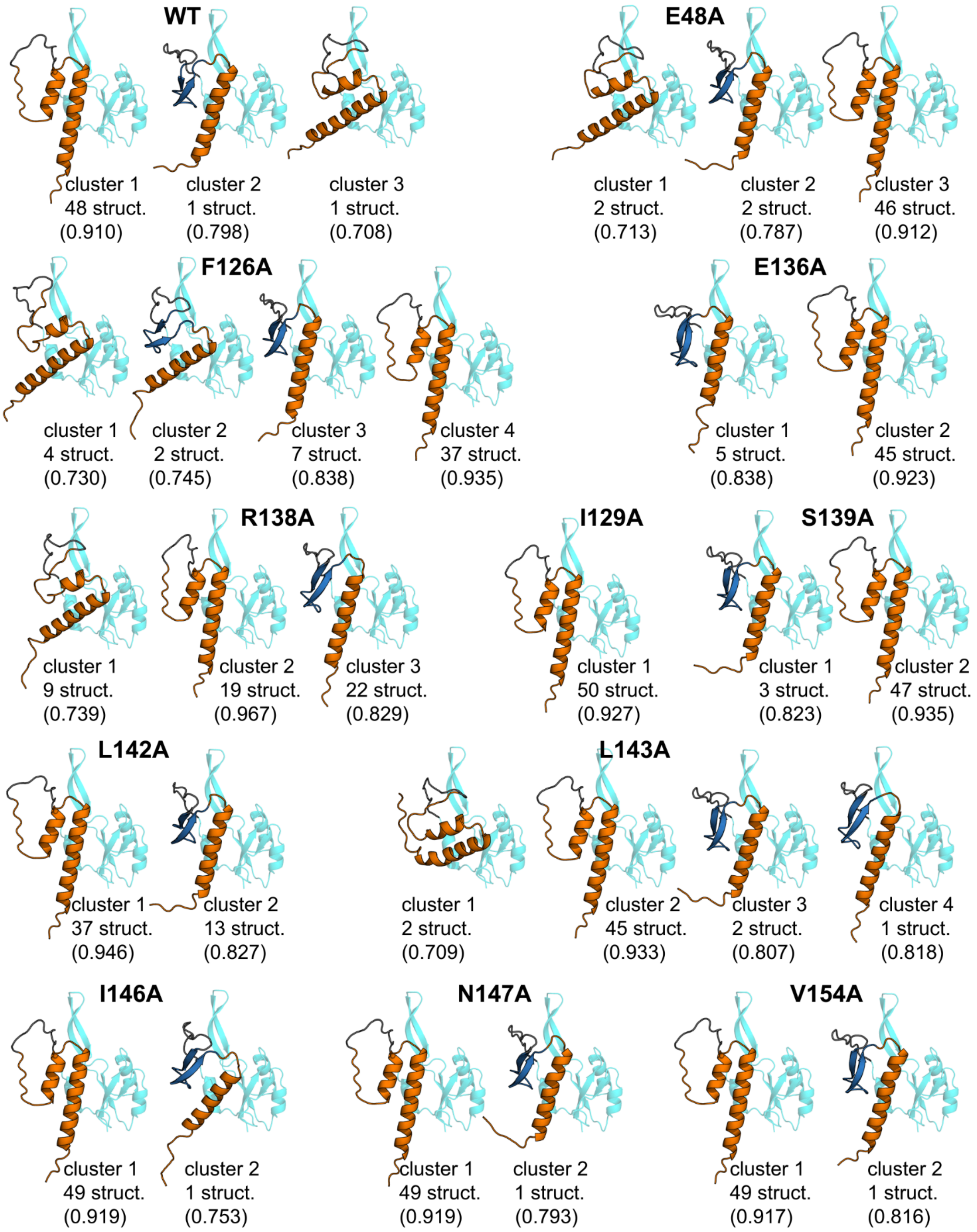

Figure S26. Representative structures of each cluster (5 model parameters, 10 seeds, 12 recycles, no dropouts, same MSA) for each RfaH variant using hierarchical clustering based on TM-score. The NTD is represented in cyan, the CTD in orange, and the β-strand CTD region (residues 114-131) in blue when present. The number of structures per cluster is indicated below. The number in parentheses is the average TM-score of each cluster against the best predicted structure (rank 1) for each variant.

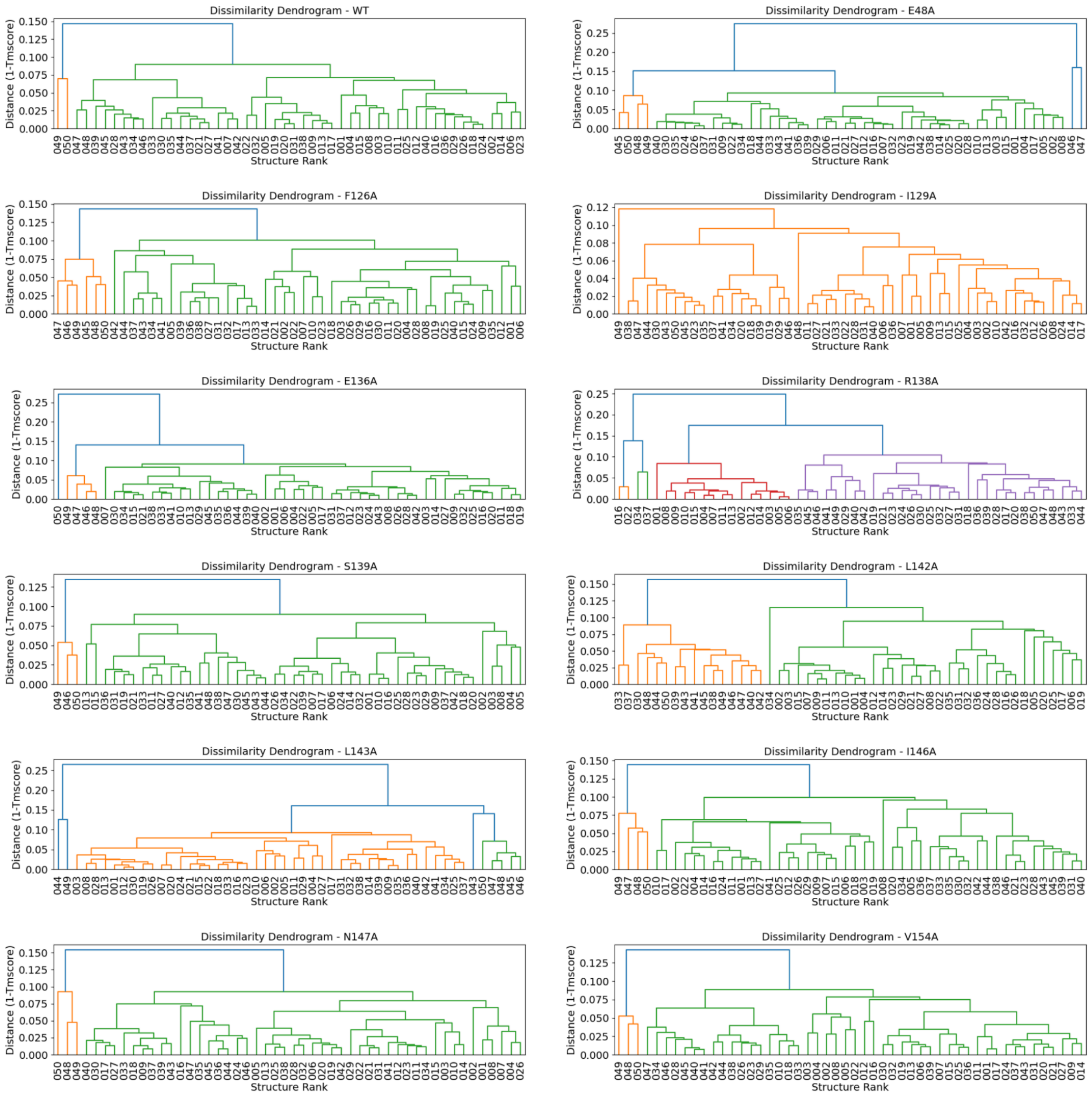

Figure S27. Hierarchical clustering based on the dissimilarity (1 - TM-score) of all predicted structures generated for all variants using ColabFold (5 model parameters, 10 seeds, 12 recycles, with dropouts, same MSA as RfaH WT) against the structure ranked as 1 for each variant. A distance of 0.12 was considered as optimal to cluster the structures in different groups based on their TM-score against the best predicted structure. The different colors in the dendrograms represent the different clusters for each variant.

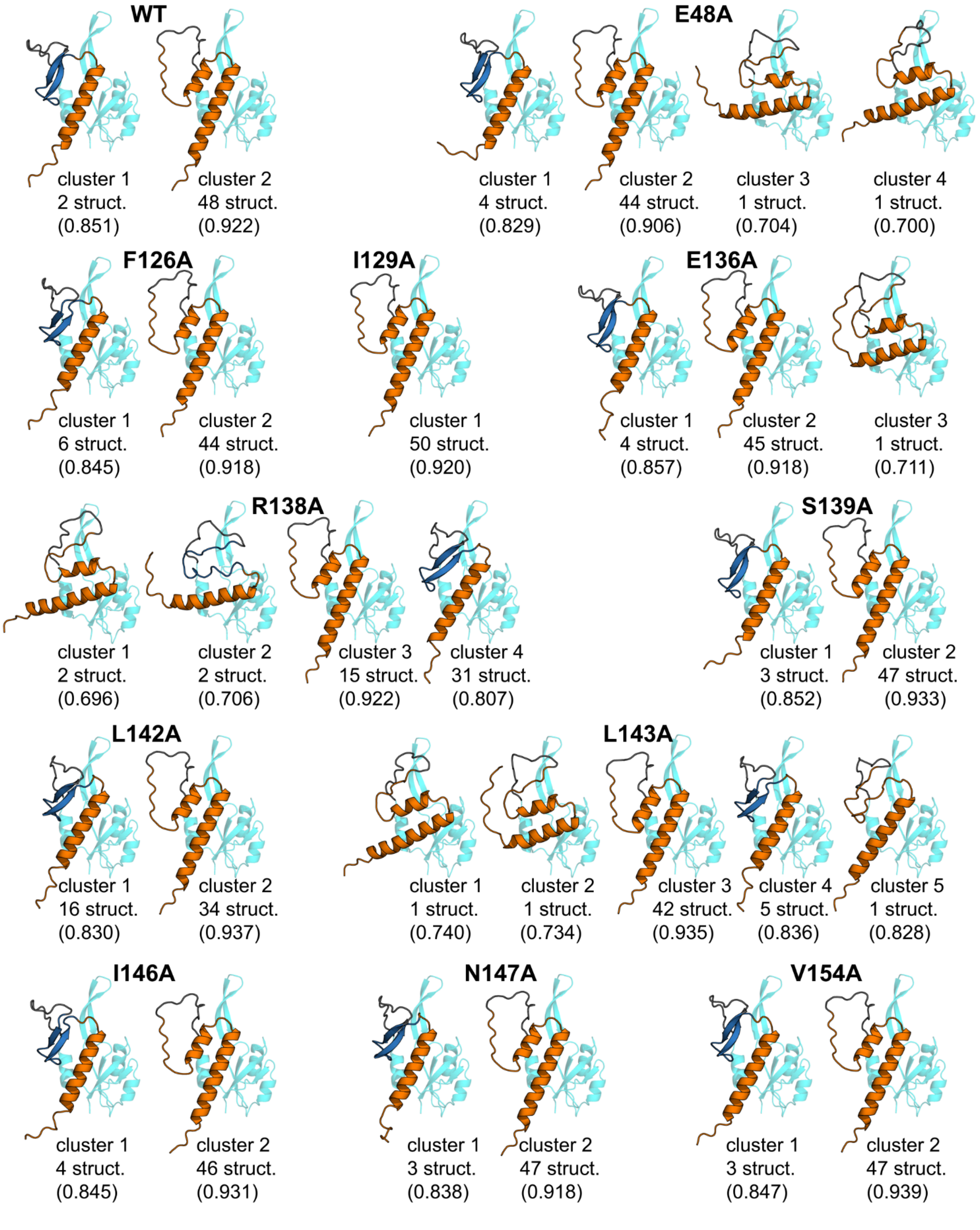

Figure S28. Representative structures of each cluster (5 model parameters, 10 seeds, 12 recycles, with dropouts, same MSA) for each RfaH variant using hierarchical clustering based on TM-score. The NTD is represented in cyan, the CTD in orange, and the β-strand CTD region (residues 114-131) in blue when present. The number of structures per cluster is indicated below. The number in parentheses is the average TM-score of each cluster against the best predicted structure (rank 1) for each variant.

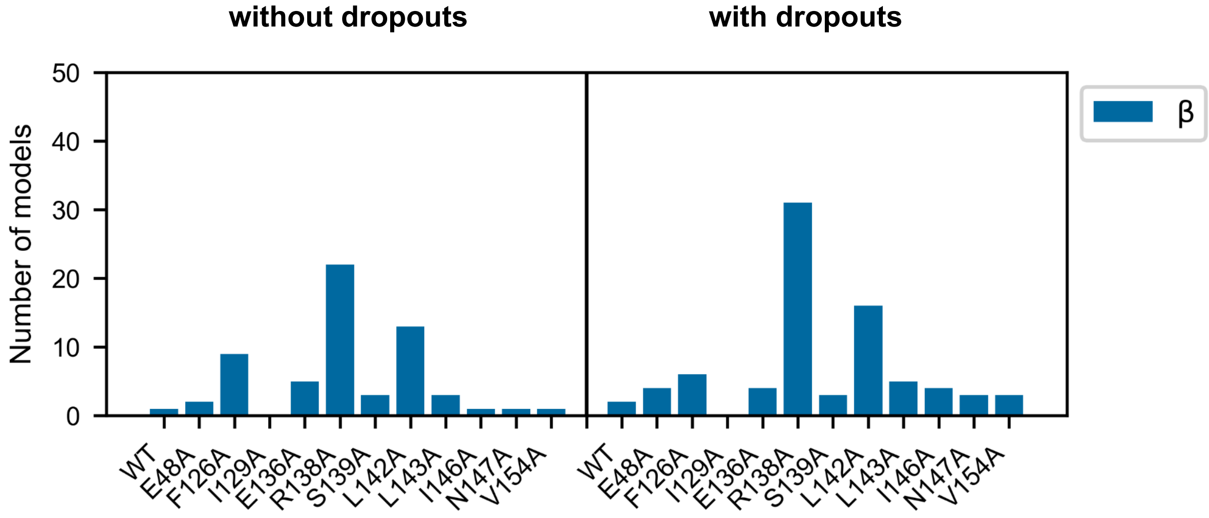

Figure S29. Counting of the number of predicted structures with β-strand (blue) content in the region 114-131 of the CTD of all RfaH variants generated using ColabFold, based on the results from hierarchical clustering. For all protein structure predictions, ColabFold was run without using structural templates, using 10 random seeds, 5 model parameters, 12 recycles, and the same MSA as RfaH WT, thus generating 50 models per input sequence, either without or with dropouts.

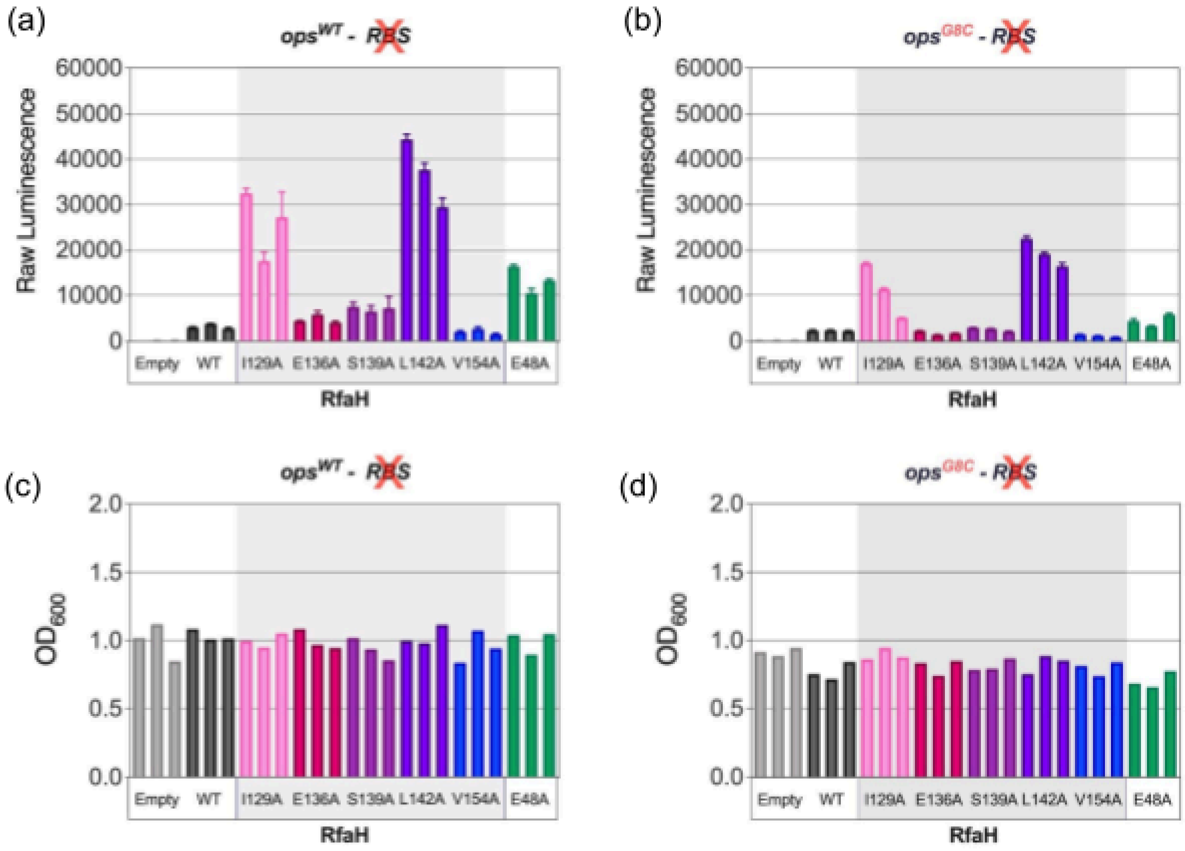

Figure S30. Raw luminescence and cell growth. RfaH variants co-transformed with *ops* variants upstream of the *luxCDABE* operon: one with *ops*^{WT} (a, c), and another with a mutation in ops (*ops*^{G8C}) (b, d). Luminescence without normalization (Raw luminescence) of each colony is shown (a, b). The graph shows the mean value and the SD of six technical replicates for each colony. The cell density (OD_{600}) measured for each colony is shown (c, d).
